# Nanobodies Enhancing Cancer Visualization, Diagnosis and Therapeutics

**DOI:** 10.3390/ijms22189778

**Published:** 2021-09-10

**Authors:** Dhaneshree Bestinee Naidoo, Anil Amichund Chuturgoon

**Affiliations:** Discipline of Medical Biochemistry and Chemical Pathology, Faculty of Health Sciences, Howard College, University of Kwa-Zulu Natal, Durban 4013, South Africa; NaidooD4@ukzn.ac.za

**Keywords:** cancer, antibodies, nanobodies, imaging, diagnosis and therapies

## Abstract

Worldwide, cancer is a serious health concern due to the increasing rates of incidence and mortality. Conventional cancer imaging, diagnosis and treatment practices continue to substantially contribute to the fight against cancer. However, these practices do have some risks, adverse effects and limitations, which can affect patient outcomes. Although antibodies have been developed, successfully used and proven beneficial in various oncology practices, the use of antibodies also comes with certain challenges and limitations (large in size, poor tumor penetration, high immunogenicity and a long half-life). Therefore, it is vital to develop new ways to visualize, diagnose and treat cancer. Nanobodies are novel antigen-binding fragments that possess many advantageous properties (small in size, low immunogenicity and a short half-life). Thus, the use of nanobodies in cancer practices may overcome the challenges experienced with using traditional antibodies. In this review, we discuss (1) the challenges with antibody usage and the superior qualities of nanobodies; (2) the use of antibodies and nanobodies in cancer imaging, diagnosis, drug delivery and therapy (surgery, radiotherapy, chemotherapy and immunotherapy); and (3) the potential improvements in oncology practices due to the use of nanobodies as compared to antibodies.

## 1. Cancer

Cancer is a complex disease, which is characterized by uncontrolled cell growth, the dysregulation of apoptosis, invasion, angiogenesis and metastasis [1,2]. Unfortunately, cancer incidence and mortality rates are drastically increasing [1]. Globally, there were approximately 18 million (mil) new cancer cases in 2018, and the cancer burden is expected to increase to 29–37 mil new cases by 2040 [1,3]. Commonly diagnosed cancers include lung, female breast, colorectal and prostate cancer [1,3]. Worldwide, cancer is responsible for one in six fatalities [1]. In 2018, 9.6 mil cancer patients succumbed to the disease, with lung cancer being responsible for round 18.4% of cancer-associated deaths [1,3].

The most prevalent malignancies in children (<15 years) are leukemias, lymphomas and brain and nervous system cancers, whereas in older patients (15–59 years), it is breast, liver and lung cancers [4,5]. In men and women (0–74 years), the risk of developing cancer is 22.4 and 18.2%, respectively [4,6]. The highest risk is for lung, breast, prostate and colorectal cancer [4,6]. The most commonly diagnosed cancers in men are lung (1.37 mil) and prostate (1.28 mil) cancers, whereas in women, it is breast (2.09 mil) and lung (0.72 mil) cancers [4,6]. In men and women (0–74 years), the risk of cancer mortality is 12.7 and 8.7%, respectively [4,6]. Pancreatic, lung, liver and intrahepatic bile duct cancers have the highest mortality rates, whereas thyroid, prostate and bladder cancers have a lower death rate [4,6]. Cancer is a serious health concern; therefore, the development of new techniques to visualize, diagnose and treat cancer is imperative.

## 2. Antibodies

In humans, antibodies (Abs)/immunoglobulins (Igs: example IgG) are produced by the immune system as a defense against foreign bodies [7]. Abs have complex and highly conserved structures comprising of two identical heavy and two identical light chains, which are linked by disulfide bonds as well as non-covalent interactions [7]. The antigen-binding site contains three loops within the variable heavy (VH) domain and three variable loops within the variable light (VL) domain [7]. For decades, Abs have been generated and used in systemic cancer therapy to inhibit growth factors or receptors that contribute to cancer progression [7]. The three mechanisms that Abs use to target cancer cells are destruction by immune cells, changes in biological processes and the delivery of cytotoxic agents [7].

Although Abs have been beneficial in visualizing, diagnosing and treating cancer, there are several limitations with the use of Abs [7]. Firstly, high levels of immunogenicity (Table 1) decrease the efficacy of Abs. In patients, murine Abs stimulate an immunogenic response resulting in human anti-mouse Ab production, which neutralizes the Abs’ function by targeting murine idiotopes [7]. In an attempt to decrease immunogenicity, chimeric Abs were developed [7]. These Abs contain human heavy and light chain regions as well as murine variable domains [7]. Chimeric Abs also stimulate an immunogenic response that generates human anti-chimeric Abs [7]. However, the immunogenicity of chimeric Abs (30%) is much lower than that of murine Abs (50–75%) [7]. Additionally, the antigen-binding VH and VL loops can be grafted into a human IgG Ab to further decrease immunogenicity [7].

The large size of conventional Abs (150 kDa and 14.2 nm × 8.2 nm × 3.8 nm dimensions) restricts its penetration into tumor tissue (Table 1) [7,8]. Previous in vivo studies have demonstrated that in one gram of solid tumor, there is approximately 0.001–0.01% of the injected Ab present, indicating the low level of penetration [7]. Additionally, poor penetration may be due to high affinity Abs binding strongly to the first antigen encountered [7].

Conventional Abs are fragile (Table 1), which limits treatment administration to intravenous or subcutaneous injections [7]. Moreover, due to the Abs’ complex structure and post-translational modifications, they are mostly expressed in mammalian cells; therefore, large-scale production is expensive (Table 1) [7].

In an attempt to overcome the limitations of monoclonal Abs (mAbs), the antigen-binding fragment (Fab, ~50 kDa), variable fragment (Fv, ~15 kDa) and single-chain Fv (scFv, ~30 kDa) were developed [8,9]. In comparison to mAbs, mAb-derived fragments are smaller in size, cleared faster and possess improved penetration capabilities [9,10]. However, the disadvantages of these fragments include decreased stability, moderate or decreased affinity, shorter serum half-life, challenges in large scale production, suboptimal tumor targeting and significant accumulation in healthy tissues (in vivo) [8,9,10]. Therefore, in an attempt to combine the beneficial characteristics of mAbs and mAb-derived fragments, diabodies (55 kDa) and minibodies (80 kDa) were developed [10].

The structure of the Abs’ VH domain includes complementarity-determining regions (CDRs) [7]. CDR1 and CDR2 contribute to the binding strength, while CDR3 contributes to antigen recognition as well as specificity [7]. The length of CDRs is a contributing factor to the antigen-interacting surface [7,8]. In Abs, the CDR3 of VH is an average length of 12 or 14 amino acids (Table 1) [7]. Thus, the antigen-interacting surface of mAbs and Ab fragments is more flat and less flexible, which restricts their interaction with the antigen surface (Table 1) [8].

Taken together, the various Ab limitations indicate a need for the development of new and alternative options that are safe and more efficient than Abs [7].

## 3. Nanobodies

Previously, functional heavy chain-only Abs were discovered in the serum of camelids [7]. In comparison to conventional Abs, heavy chain-only Abs lack light chains as well as the first constant CH1 domain; therefore, these Abs are smaller in size (95 kDa) [7,11]. Nanobodies (Nbs) are novel antigen-binding fragments (ABFs), which are produced from camelid heavy chain-only Abs [7]. These Nbs lack the heavy chain and comprise only the variable antigen-binding domain (VHH); thus, Nbs (12–14 kDa and 4 nm × 2.5 nm × 3 nm dimensions) are smaller in size than Abs [7,11]. Currently, Nbs are the smallest naturally occurring ABF that has complete binding potential [7]. The advantages of Nbs include its natural origin, reduced size, low toxicity, improved safety, water solubility, high sensitivity, stability (Table 1), antigen-binding potential and strong affinity for the target antigen [7]. Additionally, the Nbs’ physical properties include a prolonged shelf life (+4 °C and −20 °C), resistance to proteolytic degradation, tolerance to elevated temperatures (60–80 °C), increased pressure (500–750 MPa), pH changes (pH range 3.0–9.0) (Table 1) and chemical denaturants, therefore preserving the antigen-binding capacity [7]. In terms of immunogenicity, Nbs and the VH of human Ig (family III) have a sequence identity greater than 80%, indicating the low immunogenic profile of Nbs (Table 1) [7,10]. The low immunogenicity in humans allows for repeated treatment cycles [10].

Due to their small size, Nbs can penetrate deeper into tumor tissue, but Nbs also have a short half-life (Table 1), which can be a disadvantage [11]. However, Nbs can be engineered to prolong their half-life (<2 h to a few weeks) and to suit the treatment (acute or chronic) (Table 1) [12]. Additionally, Tijink et al. (2008) indicated that the Nbs’ half-life may be prolonged by fusing the Nb to an anti-albumin unit (αAlb) [13].

For effective therapy, the ideal size range for Nbs is 5–200 nm, as this may decrease elimination by renal clearance (<5 nm) as well as decrease capture by the liver and spleen reticuloendothelial system (>200 nm) [7]. This size range also allows Nbs to pass through the pores (50–200 nm) between endothelial cells, which may improve penetration [7].

In comparison to Abs, Nbs have a longer CDR3 length, which is highly involved in antigen recognition and specificity [7,8]. A longer CDR3 length allows the formation of a finger-like structure, which is able to extend into the target protein cavities and bind to unique epitopes (Table 1) [8]. Therefore, Nbs may have a higher level of interaction, recognition and specificity to the target antigen.

Upon antigen binding, the Abs’ Fc domain may stimulate Ab-dependent cellular cytotoxicity and complement-dependent cytotoxicity, which have vital roles in tumor eradication [8]. However, Nbs lack an Fc domain and are unable to trigger cytotoxicity; therefore, Nbs have been fused to an Fc domain [8].

Owing to their monomeric structure and lack of post-translational modifications, Nbs may be expressed in microbial organisms and produced in sufficient quantities, thus manufacturing costs are low while availability is high (Table 1) [7]. In addition, their monomeric structure allows Nbs to be easily formatted into different constructs (multivalent, biparatopic and bispecific molecules) (Table 1) [10,12]. A multivalent format has multiple Nbs with identical binding sites for one antigen, whereas a biparatopic format has two Nbs that bind to two different epitopes on one antigen [12]. In comparison to mAbs and bivalent mAb fragments, monovalent Nbs have a lower avidity [10]. The production of bivalent Nbs may increase avidity without dampening the beneficial pharmacokinetics and targeting capacities of Nbs [10]. Previously, bivalent Nbs have demonstrated increased avidity to their specific antigen, which improves Nb retention in target tissues [14]. The bispecific format has Nbs that bind to two different antigens [12]. In comparison to conventional Abs, these formats allow the use of Nbs in various therapeutic applications [12]. Nbs can be used as an intrabody (modulating, tracing and visualizing antigens), biomarker probes (non-invasive imaging agents) and therapeutic agents (neutralizing, receptor-ligand antagonists and effector delivery/drug therapy) [11].

Taken together, Nbs offer many beneficial properties that can greatly impact or enhance oncology practices [7].

## 4. Antibodies versus Nanobodies in Oncology Practices

The most common types of cancer treatments are chemotherapy, radiotherapy and surgery [15]. Chemotherapy is a systemic treatment able to treat cancer throughout the body, whereas surgery and radiotherapy are local treatments directed at a specific area of the body [16]. The interdisciplinary field of nanomedicine uses nanoparticles in the prevention, diagnosis and treatment of diseases at a molecular level [7]. Nanomedicine aims to develop imaging/visualization and therapeutic agents that increase efficacy and safety whilst simultaneously decreasing toxicity [7]. The term nanodiagnostics describes the use of devices with nanometer measurements for the detection of molecular events [7]. These molecular events may be used to identify and monitor diseases at an early stage [7]. Nanotherapy refers to utilizing nanoparticles to deliver drugs, as well as a treatment in order to combat diseases [7].

Current oncology practices have several limitations (high background noise, compound half-life, retention in non-target tissues, late stage diagnosis, adverse effects of treatments and low drug solubility and delivery) that translate into insufficient clinical outcomes [7]. Nanoparticles have various beneficial properties that may overcome these limitations and enhance cancer imaging, diagnosis and treatment, ultimately improving clinical outcomes [7].

### 4.1. Molecular Imaging

Cancer can be visualized using single-photon emission computed tomography (SPECT), positron emission tomography (PET), computed tomography (CT), ultrasounds and optical imaging [8,11]. Abs and Nbs may be linked to radionuclides, positron-emitting radioisotopes and near-infrared fluorophores [11]. Radionuclides (99-mTechnetium (^99^mTc), 177-lutetium (^177^Lu), 123-iodine (^123^I) and 11-iodine (^11^I)) are used in SPECT imaging, whereas positron-emitting radioisotopes (68-gallium (^68^Ga), 124-iodine (^124^I), zirconium-89 (^89^Zr) and fluor-18 (^18^F)) are used in PET imaging [11].

The interaction between programmed cell death-1 (PD-1: an immune checkpoint receptor) and its ligand PD-L1 negatively regulates T-cell responses [17]. In order to escape destruction, cancer cells increase PD-1 signaling by expressing PD-L1 [17]. In PD-L1-expressing tumors, a 111-indium (^111^In) radiolabeled PD-L1.3.1 mAb (^111^In-PD-L1.3.1) was shown to efficiently accumulate and allow the non-invasive imaging of tumors by SPECT/CT (in vivo) as early as day 1 post injection (p.i.) [18]. Chatterjee et al. (2016) also produced a radiolabeled ^111^In-PD-L1-mAb that specifically binds to tumor cells (in vitro and in vivo) and allows the detection of PD-L1-expressing tumors by SPECT/CT imaging [19]. However, the images were acquired over 1–5 days (Table 2) and there was radioactivity accumulation in healthy organs (lungs, liver, spleen and kidneys) [19].

Mesothelin is a glycoprotein that is greatly expressed in ovarian cancers and pancreatic adenocarcinomas [20]. It may be involved in many processes, such as cancer cell survival, proliferation, metastasis, invasion, tumorigenesis and chemoresistance [20]. Kobayashi et al. (2015) labeled an anti-mesothelin mAb (11–25 mAb) with 64-copper (^64^Cu) and demonstrated its high accumulation in mesothelin-expressing tumors compared to mesothelin-negative tumors as well as its potential to detect mesothelin-expressing pancreatic cancers by PET imaging (in vivo) [20].

The epidermal growth factor receptor (EGFR) has been implicated in a number of epithelial cancers (breast, lung and brain cancers) and has a role in cell growth, survival and migration [21,22]. The anti-EGFR Ab cetuximab was labeled with ^89^Zr, and ^89^Zr-cetuximab was shown to be taken up by tumors as well as visualized by PET imaging in advanced colorectal cancer patients [23]. However, there was a high accumulation of ^89^Zr-cetuximab in the normal liver, images were taken over a period of 6 days p.i., and the sixth day p.i. was the optimal scanning time point (Table 2) [23]. This may indicate that it takes 6 days for ^89^Zr-cetuximab to sufficiently accumulate in tumors and for an optimal image to be captured. In esophageal squamous cell carcinoma (ESCC), ^64^Cu-cetuximab was shown to be useful in detecting EGFR-expressing tumors using PET imaging; however, ^64^Cu-cetuximab tumor accumulation only peaked at 48 hours (h), indicating that a desired image was produced 2 days later [24].

Although Abs have shown promising results, the utilization of Abs in molecular imaging is challenging due to their large size and long serum half-life, which limits tumor penetration and increases the background-to-tumor ratio (Table 2) [9]. Moreover, capturing a desired image using radiolabeled mAbs takes a number of days [10]. The challenges with mAb probes may be solved by developing smaller mAb fragments; however, mAb fragments have mostly shown low stability, slow clearance and suboptimal tumor targeting and uptake into healthy tissues [10].

However, Nbs are ideal probes due to their various properties [8]. Nbs are stable, specific, homogenously distributed, able to rapidly accumulate in tumors, bind strongly to targets and are efficiently cleared from blood, which results in high tumor-to-background ratios (Table 2) [8,11]. Additionally, Nbs are easily conjugated to imaging agents [11].

Nuclear imaging utilizes various radionuclides and radioisotopes to visualize cancer [8]. Nbs have been produced to target cancer cell biomarkers (EGFR, human epidermal growth factor receptor 2 (HER-2) and prostate-specific membrane antigen (PSMA)) for nuclear imaging [25,26,27]. At peak time points, the absolute tumor uptake values of ^99m^Tc-labeled Nbs were lower than those of mAbs; however, Nbs produced a higher (10-fold) specific contrast [10].

Previously, HER-2 overexpression has been shown in various cancer types (breast, ovarian and colorectal cancers) and it has been related to an increased probability of cancer recurrence and aggressive tumor growth as well as poor clinical outcomes [26,28,29]. In breast cancer, SPECT imaging was used to determine the biodistribution and tumor targeting of a ^99m^Tc-labeled anti-HER-2 Nb (2Rs15d) [26]. Moreover, in mice bearing subcutaneous A431 xenografts, the biodistribution and tumor targeting of ^99m^Tc-labeled anti-EGFR Nbs were investigated by pinhole SPECT/Micro-CT [25]. Notably, both the ^99m^Tc-2Rs15d and ^99m^Tc-EGFR Nbs demonstrated high tumor uptake and rapid blood clearance but also a high renal uptake (Table 3) [25,26].

In prostate cancer, PSMA is overexpressed, which is related to castration-resistant prostate and decreased androgen-receptor expression as well as a poor prognosis [27,30]. Evazalipour et al. (2014) evaluated the tumor targeting of radiolabeled PSMA Nbs in LNCaP (PSMA+)- and PC3 (PSMA−)-xenografted mice using SPECT/Micro-CT [27]. The ^99m^Tc-PSMA30 Nb demonstrated high absolute tumor uptake, high tumor-to-normal organ ratios (Table 3) and specific binding to LNCAP cells instead of PC3 cells, indicating the potential to visualize prostate cancer (Table 2) [27].

The transmembrane glycoprotein macrophage mannose receptor (MMR) is expressed on macrophages and plays a role in tumor growth, metastasis and immune suppression [10]. Previously, ^99m^Tc-labeled anti-MMR Nbs were produced for non-invasive imaging of tumor-associated macrophages (TAM) and was shown to successfully target as well as image TAM subpopulations in vivo (Table 3) [14].

Multiple myeloma (MM) is a hematological cancer of plasma cells and is characterized by monoclonal protein (M protein) production [10]. In patients, the M protein is secreted into the bloodstream and associates with the MM cell surface [10]. The 5T2 MM model resembles human MM (clinically and biologically), and 5T2 MM cells produce a paratope of the M protein [31]. Nbs were produced against this paratope of the M protein [31]. The anti-5T2MM-idiotype Nb (R3B23) was labeled with ^99^mTc and allowed the imaging of 5T2 MM cancer progression by SPECT/Micro-CT [31]. Additionally, in minimal residual disease, these Nbs are capable of monitoring the progression of the disease in vivo (Table 3) [31].

In comparison to SPECT, clinical PET offers higher image resolution and quantitative properties; therefore, the development of PET tracers may be beneficial [10]. PET radioisotopes are favorable due to Nbs having a short half-life and can be effective in tumor detection/imaging [10,32]. Thus, an anti-EGFR Nb [32] and an anti-HER-2 Nb (2Rs15d) [29] were radiolabeled with ^68^Ga. In nude mice bearing A431 xenografts, the ^68^Ga-EGFR-Nb showed a high tumor uptake as well as high tumor-to-normal tissue ratios (Table 3) [32]. In HER-2+ tumors, ^68^Ga-NOTA-2Rs15d showed fast, specific uptake and high tumor-to-blood ratios, leading to high-specific contrast PET/CT images with no observed toxicity (Table 3) [29]. 

Ultrasound waves can be used for tumor imaging [11]. Microbubbles (µBs) are small gas-filled particles able to scatter ultrasound and are used as contrast agents [33]. Nbs can be coupled with µBs to form targeted µBs, which may improve specificity and contrast [33]. In cancer, vascular cell adhesion molecule-1 (VCAM-1) has been associated with tumor angiogenesis and metastasis [34]. Hernot et al. (2012) produced µBs coupled with an anti-VCAM-1 Nb (μB-cAbVCAM1-5) and demonstrated the imaging of μB-cAbVCAM1-5 in MC38 tumor-bearing mice using a contrast-specific ultrasound imaging mode (Table 3) [33]. The ultrasound image was used to measure the echo intensity in the region of interest (tumor), and the μB-cAbVCAM1-5 resulted in an increase in the signal [33]. Additionally, Fan et al. (2015) produced ultrasonic nanobubbles (nBs) coupled with anti-PSMA Nbs to image prostate cancer (Table 3) [35]. Using ultrasound imaging, the anti-PSMA nBs were shown to target PSMA+ cells and animal xenografts (LNCaP, C4-2 and MKN45) [35].

Optical tumor imaging involves conjugating Abs or Nbs to near-infrared dyes (NIR) or fluorophores (IRDye800CW) [11]. An example of the use of Abs in optical imaging is the anti-PD-L1-mAb. In human tumor xenografts, the NIR-PD-L1-mAb conjugate was shown to specifically bind to tumors and allow the detection of PD-L1-expressing tumors by optical imaging (in vivo) [19].

During hypoxic conditions, anti-carbonic anhydrase IX (CAIX) is upregulated and can be used to target tumor cells [36]. Van Brussel et al. (2015) produced an anti-CAIX Nb, conjugated it to IRDye800CW and demonstrated the use of the anti-CAIX Nb-IRDye800CW conjugate in the imaging of pre-invasive breast cancer (Table 3) [36]. An anti-EGFR Nb (7D12) was also conjugated to IRDye800CW, and, in a mouse model, the 7D12-IRDye800CW conjugate was shown to clearly image orthotopic tongue tumors as well as cervical lymph node metastases (Table 3) [37].

Previously, Oliveria et al. (2012) conjugated 7D12 and cetuximab to IRDye800CW [38]. In mice bearing A431 human tumor xenografts, the tumor uptake of 7D12-IRDye800CW was significantly higher than that of cetuximab-IRDye800CW [38]. Additionally, tumors were rapidly and clearly visualized following 7D12-IRDye800CW administration (30 min p.i.), whereas tumor visualization following cetuximab-IRDye800CW administration required much more time (Table 2) [38].

Taken together, Nbs have shown vast potential in the molecular imaging of cancer and may be more efficient than Abs.

### 4.2. Diagnosis

Combining a therapeutic compound with a diagnostic radiotracer and conducting a diagnostic scan allows the visualization of the compound accumulation in cancer cells [10]. Radiolabeled Nbs can be useful in identifying specific tumor-associated biomarkers, which can assist in cancer diagnosis and in determining the appropriate treatment [10]. Nbs diffuse quickly into tissue and specifically accumulate in target tissues (minimal accumulation in non-target tissues), which results in the rapid production of high-contrast SPECT or PET images (1–3 h), therefore allowing early patient diagnosis (Table 2) [10]. Additionally, radiolabeled Nbs can be used for a whole body scan that detects a specific biomarker in primary tumors and metastasized lesions [10]. In comparison to blind biopsies (standard oncology practice), a scan of the entire body may provide extensive information on the presence of malignant cells/tissues throughout the patient [10]. Notably, diagnostic scans aid in estimating dosage, monitoring treatment responses, evaluating treatment efficiency and anticipating the adverse effects of treatments [10]. Xing et al. (2019) generated a ^99m^Tc-labeled anti-PD-L1 single-domain Ab (sdAb) (NM-01) and demonstrated its imaging and diagnostic potential using SPECT/CT [39]. In non-small cell lung cancer (NSCLC) patients, molecular imaging using ^99m^Tc-NM-01 was considered a safe diagnostic procedure that allowed the imaging of PD-L1-expressing cells without causing any adverse effects [39]. Additionally, in PD-L1-positive cancer patients, ^99m^Tc-NM-01 SPECT/CT imaging can potentially monitor PD-L1 expression, which will aid in the diagnosing and staging of patients (Table 3) [39].

### 4.3. Surgery

In 2015, approximately 80% of new cancer cases required surgery [40]. Worldwide, about 45 mil surgical procedures will be required by 2030; however, a small percentage of cancer patients are able to receive safe, inexpensive and well-timed surgery [40]. Surgery is a fundamental treatment option used to prevent, diagnose and cure cancer [40]. Additionally, surgery is used for palliative care and cosmesis [40]. Preventive surgery aims to remove tissue that is susceptible to carcinogenesis, while diagnostic techniques are important in determining the appropriate treatment and management of cancer [40]. In the attempt to cure cancer patients, surgery (minor or major) is vital, always needed and greatly effects patient outcomes [40]. Resection and reconstructive surgery is important for palliative care and improving cosmesis, respectively [40]. In 2030, surgery will be required by approximately 80% of the estimated 21.6 mil new cancer cases [40]. A promising use of Nbs in surgery is imaging-guided surgery [11]. Previously, the anti-HER-2 Ab trastuzumab was dual labeled with ^111^In and IRDye800CW [41]. Deken et al. (2019) demonstrated that in orthotopic HER-2+ BT474 tumors, ^111^In-DTPA-trastuzumab-IRDye800CW specifically accumulated as well as allowed the visualization of tumors by micro-SPECT/CT and fluorescence imaging [41]. Additionally, the dual-labeled trastuzumab enabled the image-guided resection of tumors [41].

Kijanka et al. (2013) conjugated an anti-HER-2 Nb (11A4) to IRDye800CW and demonstrated that 11A4-IRDye800CW increased tumor accumulation and tumor-to-background ratios as compared to trastuzumab-IRDye800CW in human xenograft breast cancer models (Table 2) [42]. Additionally, 11A4-IRDye800CW was used in image-guided surgery for the removal of SKBR3 tumors in mice (Table 3) [42].

Both Abs and Nbs have showed potential in image-guided surgery. However, Nbs may be the better option due to the high tumor-to-background ratios as well as high and rapid tumor accumulation, which may allow a more precise visualization of the tumor borders for resection (Table 2).

### 4.4. Radiotherapy

Radiation therapy remains an important and highly cost-effective cancer treatment [43]. It is administered to about 50% of cancer patients and contributes to curative treatment by approximately 40% [43]. Radiotherapy aims to expose malignant cells to the maximum radiation dose while minimally exposing normal cells [43]. Radiation damages DNA, which may block cell proliferation and induce cell death [43]. Fortunately, healthy cells are able to quickly repair themselves, survive and function normally [43]. However, cancer cells are less efficient at repairing themselves, which results in their destruction [43]. Radiotherapy can be used in combination with surgery, chemotherapy and immunotherapy [43]. For example, radiation used before surgery aims to reduce tumor size, whereas radiation used after surgery aims to destroy the remaining microscopic tumor cells [43]. The delivery of radiation to cancerous cells may be carried out externally or internally [43]. The most common radiation delivery method is exposing the tumor location (target area) to an external high-energy X-ray beam [10,43]. Internal radiation is delivered by radioactive sources, directly into the tumor location [43]. Although radiotherapy is beneficial, there are several adverse effects, for example, oral mucositis, hepatotoxicity and nephrotoxicity [44].

Targeted radionuclide therapy (TRNT) can be used to deliver radiation to malignant cells while minimally effecting healthy cells [10]. There are two types of TRNT agents: (1) those that naturally accumulate in malignant tissues (131-iodine—(^131^I), ^131^I-MIBG and 89-strontium (^89^Sr) chloride) and (2) those that target tumor-associated antigens expressed on the cell surface [10]. Linear energy transfer (LET) is defined as the energy (radiation) released over a certain distance [10]. At a particular absorbed dose, cytotoxicity is greater at high LET as compared to low LET radiation [10]. TRNT has been investigated using β-emitting radioisotopes (yttrium-90 (^90^Y), ^131^I and ^177^Lu) that have a low LET and α-emitting radioisotopes (212-bismuth (^212^Bi), 225-actinium (^225^Ac)) that have a high LET value [10]. Low LET radiation induces limited ionization and DNA damage, whereas high LET induces clusters of DNA damage [10]. The α-emitting radioisotopes are ideal for treating small cell burdens as they have a short path length; therefore, energy is deposited to a few cells [10]. The Food and Drug Administration (FDA) approved the first α-emitting radioactive agent 223-radium (^223^Ra) chloride for castration-resistant prostate cancer as well as symptomatic bone metastases in 2013 [10].

Radioimmunotherapy (RIT) is a term used to describe the use of mAbs to transport toxic radiation to a specific location [10]. TRNT has been studied using mAbs, for example, ^177^Lu-trastuzumab and ^177^Lu-cetuximab [10]. Rasaneh et al. (2010) demonstrated the high cytotoxicity of ^177^Lu-trastuzumab in HER-2-expressing cells [45]. Notably, ^177^Lu-trastuzumab induced a fivefold higher level of toxicity than the unlabeled trastuzumab [45]. In ESCC, ^177^Lu-cetuximab was shown to inhibit tumor growth [24], demonstrating its potential in RIT.

Lymphomas are highly radiosensitive; therefore, an effective treatment requires a low absorbed dose [10]. CD20 is expressed in 90% of B-cell non-Hodgkin’s lymphoma [46]. Previously, radiolabeled anti-CD20 mAbs (^90^Y-ibritumomab tiuxetan and ^131^I-tositumomab) was approved by the FDA for low-grade B-cell non-Hodgkin’s lymphoma treatment [10]. Notably, 80% of patients have been shown to respond to anti-CD20 RIT [10]. Blood-borne cancers respond positively to RIT; however, epithelial tumors (colorectal, breast, prostate, ovarian and pancreatic tumors) have shown limited success [10]. This can be explained by epithelial-derived carcinomas being less radiosensitive, thus requiring a higher absorbed dose [10].

The dose reaching the tumor decreases as the tumor radius increases; therefore, RIT is more suitable for metastatic cancer [10]. In colorectal cancer patients with liver metastases, radiolabeled anti-carcinoembryonic antigen Ab (^131^I-labetuzumab) treatment seemed to improve overall survival and five-year survival, suggesting the potential of RIT as an adjuvant therapy in metastatic disease [47]. In glioblastoma multiforme, conventional therapies (radio and chemo) are not effective due to the high doses being toxic to healthy brain tissue [10,48]. After surgery, the administration of radiolabeled anti-tenascin mAb 81C6 directly into the resection cavity resulted in an overall survival of 64 weeks as compared to 52 weeks for conventional therapies [48].

In large carcinomas, the mAb pharmacokinetic properties may limit RIT success [10]. Due to the large mAb size and physiological barriers, a small percentage of injected mAb penetrates into the tumor, while a higher percentage of mAb remains in the blood and accumulates in other tissues [10]. The mAb affinity affects tumor penetration; thus, high-affinity mAbs remain in the perivascular region, while lower affinity mAbs penetrate deeper [10]. However, leaky vasculature and lymph drainage impairment result in a greater accumulation of mAbs in tumors as compared to smaller molecules [10]. Moreover, the prolonged blood residence time (BRT) of mAbs may increase toxicity in healthy tissues and decrease radioactivity accumulation in tumor tissues [10]. In RIT, murine mAbs are often used instead of humanized mAbs in order to decrease BRT [10]. Due to the weaker interaction between murine mAbs and human Fc receptors, murine mAbs are cleared faster, thus decreasing BRT [10]. However, the use of murine mAbs leads to human anti-murine Ab production, which limits repeated dosing [10]. To increase tumor penetration as well as fasten blood and healthy tissue clearance, mAb-derived fragments have been developed; however, the fragments have disadvantages (as stated earlier) [10]. Diabodies (55 kDa) and minibodies (80 kDa) have shown potential in TRNT [10].

**Figure 1:**
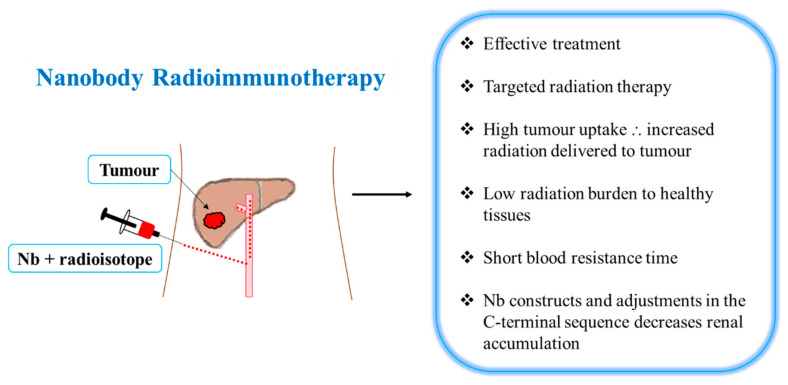
The potential of nanobody’s in radioimmunotherapy.

Nbs possess many characteristics (as mentioned earlier) that may be beneficial in RIT [7]. A camelid sdAb fragment and rituximab (anti-CD20 Ab) were radiolabeled with ^177^Lu for targeted therapy [46]. Despite the fact that ^177^Lu-DTPA-rituximab delivered more radiation to the tumor than ^177^Lu-DTPA-sdAb-9079, ^177^Lu-DTPA-sdAb-9079 was shown to be as effective as the ^177^Lu-DTPA-rituximab treatment in mice (similar median survival) [46]. However, ^177^Lu-DTPA-sdAb 9079 delivered low levels of radiation to healthy organs (except the kidneys) (Figure 1 and Table 3), whereas ^177^Lu-DTPA-rituximab delivered much higher radiation doses to healthy organs (blood, bone and spleen) [46]. More recently, Dekempeneer et al. (2020) labeled an HER-2 Nb (2Rs15d) with 213-bismuth (^213^Bi) and demonstrated its high tumor uptake, low healthy tissue uptake and increased median survival in mice (Table 3) [49].

Radiolabeled Nbs are characterized by increased renal clearance leading to moderate absolute tumor uptake and high renal accumulation (limiting factor), which affects TRNT efficiency [10]. Producing bivalent or multivalent Nb constructs as well as elevating the hydrodynamic radius of molecular vectors may aid in increasing absolute tumor uptake [10]. Previously, the fusion of an albumin-binding domain (ABD) to a bivalent anti-EGFR Nb increased BRT and tumor uptake while decreasing renal retention [13]. In A431 tumor-xenografted mice, tumor-to-kidney ratios were higher for the bivalent anti-EGFR Nb fused to an ABD compared to the bivalent anti-EGFR Nb [13]. However, the albumin-binding affinity can increase radiation exposure to healthy tissues as well as extend BRT [10].

In the renal tubuli, the charge-based interactions between proteins and the megalin/cubuilin system result in the renal retention of small proteins [10]. The kidney retention of Nbs is mostly due to the polar residues in the Nbs’ C-terminal amino acid sequence [50]. Studies have indicated that the renal retention of Nbs can be decreased by adjusting the Nb C-terminal sequence (Figure 1) [10]. Adjustments in the C-terminal sequence may be achieved by co-administration with gelofusine and/or positively charged amino acids [10]. In CD-1 mice, the co-administration of a gelofusine and L-lysine decreased renal uptake and improved the tumor uptake of a ^99m^Tc-labeled anti-EGFR Nb (7C12) [51]. In comparison to Nbs with a highly charged C-terminal amino acid tag (Myc-His-tagged), the administration of untagged ^177^Lu-labeled anti-HER-2 Nb (2Rs15d) co-infused with gelofusine resulted in a decrease in renal retention (95%) while retaining tumor-targeting capacity [50]. In HER-2+ SKOV3 tumor-xenografted mice, TRNT was performed using the untagged ^177^Lu-DTPA-2Rs15d co-infused with gelofusine [50]. Notably, the dosimetry calculations demonstrated that the doses delivered to the tumor and kidneys were higher than the doses delivered to healthy tissues (Table 3) [50]. In comparison to the untagged ^177^Lu-DTPA-2Rs15d, the ^177^Lu-DTPA-trastuzumab delivered six times more radiation to the tumor; however, it also delivered a very high (26–4180-fold) radioactivity burden to healthy organs [50]. In mice bearing small established HER-2 + tumors, Nb-based TRNT resulted in a tumor growth blockade and a substantial event-free survival (Table 3) as compared to the control groups [50]. Kidney samples revealed no evidence of renal inflammation and cell death; however, signs of toxicity in kidneys should be investigated beyond 6 months, as kidneys are late-response tissues [50].

In RIT, mAbs strongly target the tumor; however, an elevated radiation dose is delivered to heathy tissues [10]. Therefore, the administration of a lower radiolabeled mAb concentration is needed to limit the toxicity to healthy tissues, which results in a lower radioactive dose reaching the tumor [10].

In micro-metastatic and minimal residual disease, Nb-based TRNT will be useful due to the Nbs’ capacity to deliver radiation to specific targets [10]. Combining a Nb and an α-emitter may specifically deliver increased toxicity to micro-metastasized cancer cells, while minimally affecting healthy cells [10]. In comparison to mAbs, Nbs have enhanced tumor penetration, which will aid in treating solid tumors [10]. However, the impact of Nb-based TRNT in treating solid tumors requires further research [10].

### 4.5. Drug Delivery

Abs can target specific tumor antigens; thus, conjugates comprising an Ab and a drug have been produced to deliver cytotoxic drugs/agents to antigen-expressing cells and to enhance the drug/agents’ therapeutic index [28]. Trastuzumab has been shown to inhibit HER-2 signaling and induce Ab-dependent cellular cytotoxicity [28]. Previously, trastuzumab has been conjugated to the cytotoxic agent DM1 (T-DM1) in order to specifically target HER-2-expressing cells [52]. In HER-2+ tumor models, T-DM1 administration led to selective and specific anti-tumor effects [52]. In addition, T-DM1 resulted in mild toxicity and significant clinical activity in HER-2+ metastatic breast cancer patients [28]. Inhibitors of topoisomerase I induce DNA breaks and apoptotic cell death [53]. Ogitani et al. (2016) conjugated an anti-HER-2 Ab to a topoisomerase I inhibitor (DS-8201a) and demonstrated the potent anti-tumor activity of DS-8201a in HER-2+ models [53].

Immunotoxins are made up of an Ab linked to a toxin, and they specifically target tumor cells for destruction [54]. The immunotoxin moxetumomab pasudotox was produced by combining the anti-CD22 mAb Fv fragment with a Pseudomonas exotoxin A fragment [55]. Kreitman et al. (2012) demonstrated that in chemotherapy-resistant hairy cell leukemia, moxetumomab pasudotox administration led to high overall response rates as well as complete remission in 46% of patients [56].

Nbs can be used to deliver cargo (toxins/drugs) specifically to tumors, which will decrease the side effects and toxicity to normal cells [11]. Abs have an Fc tail that leads to Fc-mediated clearance or triggers an immune response, which may inhibit cargo delivery [11]. In comparison to conventional Abs, Nbs lack an Fc tail, which is an advantage in drug delivery [11].

Immunotoxins have also been generated using Nbs. Previously, vascular endothelial growth factor receptor-2 (VEGFR2) was shown to be overexpressed and dysregulated in various cancers (lung, colon and breast cancers) [8]. VEGFR2 is involved in stimulating tumor angiogenesis and outgrowth [8]. Behdani et al. (2013) produced an immunotoxin by linking a truncated form of Pseudomonas exotoxin A (PE38) to an anti-VEGFR2 Nb and showed the immunotoxin was able to inhibit the proliferation of VEGFR2-expressing cells (Table 3) [54]. Moreover, an anti-EGFR Nb-cucurmosin immunotoxin was shown to inhibit the cell viability of EGFR-expressing tumor cell lines as well as induce HepG2 and A549 apoptosis (Table 3) [57]. More recently, Zhang et al. (2020) demonstrated that a bispecific anti-EGFR-Nb-cucurmosin immunotoxin selectively kills cancer cells through apoptosis (Table 3) [58]. Cao et al. (2020) produced three anti-HER-2 immunotoxins by linking anti-HER-2 sdAb to the PE24X7 toxin [59]. The use of sdAb allowed these immunotoxins to be efficiently expressed, highly soluble and stable [59]. The anti-HER-2-PE24X7 immunotoxins showed high selectivity and cytotoxicity in HER-2+ tumor cells [59]. Additionally, tumor growth was efficiently inhibited by one anti-HER2-PE24X7 immunotoxin in vivo (Table 3) [59]. These studies demonstrate the potential of combining Nbs with toxins for the treatment of tumors [58,59].

**Figure 2:**
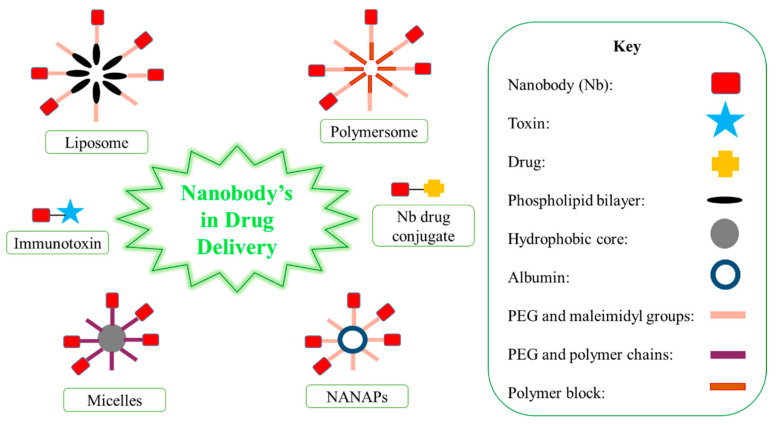
The potential of nanobodies in drug delivery.

Nbs have been used to encapsulate drugs, which improves solubility, stability and the rate of clearance [11]. There are various Nb carriers, including liposomes, micelles, albumin-based nanoparticles (NANAPs) and polymer-based polymersomes/polyplexes (Figure 2) [11]. The PEGylation process may be performed on these carries, and this process extends blood half-life [11]. At the end of the PEG block, maleimidyl groups and polymer chains enable Nb conjugation to the carriers [11]. The conjugation of Nbs on these carriers allows for selective targeting [11]. Liposomes are located on lipid bilayers and encourage the intracellular delivery of drugs, whereas micelles and NANAPs have a hydrophobic core that allows for drug encapsulation [11]. Synthetic polymer-based particles include polymersomes [11]. Nb carriers are used to protect healthy tissues from drugs/toxins administered systemically, increase the solubilization of hydrophobic drugs, and increase the drug concentration in one dose, subsequently decreasing the number of repetitive doses [8].

For example, Oliveira et al. (2010) conjugated an anti-EGFR Nb (EGa1) to liposomes (EGa1-L) and demonstrated that EGa1-L decreased EGFR levels on the cell membrane due to receptor internalization as well as mediated EGFR sequestration resulting in receptor degradation [60]. Additionally, EGa1-L was shown to inhibit tumor cell proliferation (in vitro) and downregulate EGFR (in vivo) (Table 3) [60]. Notably, an anti-EGFR scFv-liposome conjugate was unable to produce a downregulatory effect [60].

The insulin-like growth factor 1 receptor (IGF-1R) kinase inhibitor (AG538) was encapsulated in EGa1-decorated liposomes [61]. The AG538-loaded EGa1 Nb-liposomes lead to efficient intracellular AG538 delivery, an EGFR blockade, IGF-1R stimulation and the inhibition of tumor cell proliferation (Table 3) [61].

Previously, polymeric micelles were decorated with the EGa1 Nb and labeled with rhodamine [62]. The results demonstrated that in A431 and UM-SCC-14C cancer cells (EGFR overexpressing), there was high binding and uptake of the rhodamine-labeled EGa1-micelles through interaction with the EGF receptor [62]. In addition, EGa1 Nb polymeric-micelles inhibited tumor growth (in vivo) (Table 3) [63].

The epithelial cells of various organs express the c-Met receptor during embryogenesis as well as adulthood [12]. The activation of c-Met in tumor cells stimulates various signaling pathways, which results in cell growth, invasion, metastasis and the evasion of apoptosis [12]. Heukers et al. (2014) produced anti-Met Nbs conjugated to NANAPs (anti-Met-NANAPs) [64]. The results revealed that in Met-expressing cells, there was binding, uptake and lysosomal degradation of anti-Met NANAPs. In addition, anti-Met NANAPs downregulated total Met protein in tumor cells (Table 3) [64].

Altintas et al. (2013) encapsulated multikinase inhibitor 17,864 in Ega1-coated NANAPs and demonstrated that the particles were internalized and digested, that the kinase inhibitor was released in the cell, and the proliferation of cancer cells was decreased (in vitro) (Table 3) [65].

Taken together, Nbs have been shown to be beneficial in drug delivery.

### 4.6. Chemotherapy

Chemotherapy is described as the chemical treatment of disease [15]. The treatment of cancer using chemotherapeutic drugs began in the 1930s [15]. Chemical agents/drugs are used to destroy rapidly growing cancerous cells [15]. Importantly, chemotherapeutic drugs are aimed to attack a greater proportion of cancer cells than normal cells; however, a larger number of healthy cells are affected [66]. Although chemotherapy is mostly beneficial, there are adverse effects, including fatigue, hypersensitivity, gastrointestinal tract (nausea, vomiting, mucositis, diarrhea and constipation), hematological (anemia), neurological (cognitive impairments, neuropathy and neurotoxicity), hepatic, and renal (renal dysfunction) effects, and the risk of cardiovascular disease [67]. Chemotherapeutic drugs can be conjugated to an Ab/Nb in order to increase the drugs’ tumor specificity and protect healthy tissues from drug toxicity.

Ding et al. (2019) demonstrated the use of trastuzumab (Tra)-decorated polymersomes (Ps) for the targeted delivery of epirubicin hydrochloride (EPI·HCL) to HER-2+ SKOV-3 ovarian tumors [68]. The EPI·HCL-loaded Tra-Ps (Tra-Ps-EPI) was able to deliver substantial amounts of EPI·HCL to cells, which induced a higher level of cytotoxicity than non-targeted Ps-EPI (in vitro) [68]. Additionally, in SKOV-3 ovarian tumor-bearing nude mice, Tra-Ps-EPI demonstrated deep tumor penetration and a greater tumor growth inhibition than that of free EPI·HCL as well as non-targeted Ps-EPI [68]. Another example is in EGFR-overexpressed cell lines, doxorubicin loaded in cetuximab–DNA conjugates was shown to induce a greater level of cytotoxicity than that of free doxorubicin [69].

Nbs have also been used to transport chemotherapeutic drugs, for example, EGFR and PSMA Nbs. Previously, Talelli et al. (2013) encapsulated doxorubicin in EGa1 Nb polymeric-micelles and demonstrated that doxorubicin-EGa1-Nb polymeric-micelles more effectively inhibited tumor growth (Table 3) than EGa1-Nb polymeric-micelles [63]. More recently, Rosenfeld et al. (2020) isolated anti-PSMA Nbs and conjugated an anti-PSMA Nb (NB7) to doxorubicin [30]. The NB7-doxorubicin conjugate was shown to accumulate in PSMA+ tumors and specifically internalize in PSMA+ cells, leading to doxorubicin-induced cytotoxic activity (Table 3) [30]. Although the NB7-doxorubicin conjugate dosage was extremely lower than the doxorubicin dosage, the Nb conjugate induced a similar level of tumor growth inhibition (in vivo) as that of doxorubicin [30]. This demonstrates the high efficacy of the NB7-doxorubicin conjugate at a low dosage [30]. Additionally, a low dosage may decrease the exposure of healthy tissues to drug toxicity.

Although both Abs and Nbs have been investigated as drug carriers and have shown positive results, the properties of Nbs may allow Nbs to be more effective than Abs. For example, the specificity and fast clearance of Nb drug carriers may allow for a substantial reduction in drug toxicity to healthy tissues.

### 4.7. Immunotherapy

**Figure 3:**
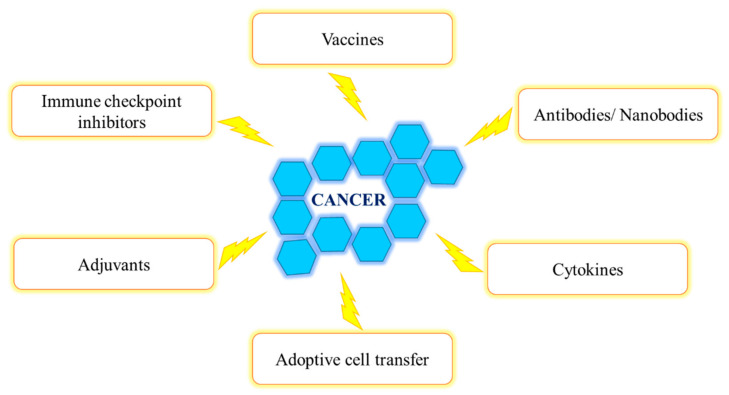
Immunotherapeutic strategies for cancer.

Cancer immunotherapy began in 1908 after the immune systems’ role in surveilling and eradicating cancerous cells was postulated by Paul Ehrlich [70]. Thereafter, studies conducted by William Coley indicated the efficacy of the immune system to combat tumors [70]. The ability of the immune system to identify and eliminate tumor cells can lead to cancer remission [70]. Immunotherapy aims to treat cancer by activating and enhancing the body’s anti-cancer immune responses as well as combining the immune defense with external therapy (chemo, radio and surgery) in order to maximize treatment efficiency [70]. There are various immunotherapeutic strategies to combat cancer (Figure 3) [70].

The process of cancer immune editing involves the elimination, equilibrium and escape stages [70,71]. During elimination, tumor development is inhibited by immune surveillance, which identifies and destroys transformed cells [70,72]. Equilibrium refers to the coexistence of an aggressive environment due to an immune response and continuous tumor cell proliferation [70]. This prevents increased tumor size; however, it eventually promotes the development of cells capable of evading immune surveillance [70,73]. In the “escape” stage, malignant cells proliferate uncontrollably and evade the immune system as well as cell death, ultimately metastasizing to other body parts [70,73]. Cancer immunotherapy strategies attempt to enhance immune responses that target cancer as well as encourage elimination while avoiding equilibrium and escape [70].

Cancer vaccines are aimed at stimulating/restoring the immune response, delaying tumor growth and shrinking tumor size; thus, vaccines can be used in the prevention and treatment of cancer [70,74]. Adjuvants aid in the modulation and enhancement of immune responses [70]. In active cancer immunotherapy (the stimulation of the host’s defense system), adjuvants in combination with vaccines increase antigen-specific reactions and improve efficacy [70]. Another cancer immunotherapy approach is ACT, which utilizes tumor-infiltrating lymphocytes (TILs) or T-cells with chimeric antigen receptors (CARs) [70]. This involves the isolation of specific cell populations from a resected tumor of one patient [70]. The TILs with the highest cancer cell affinity are isolated and grown [70]. Thereafter, these TILs are injected into the patient, usually along with interleukin (IL)-2, in an attempt to eradicate the tumor and overcome immune-suppressive signals [70].

Previously, an anti-CD33 scFv was used to produce CAR T-cells that target CD33 (CART33) [75]. In acute myeloid leukemia (AML) xenografts, CART33 therapy eradicated leukemia and prolonged disease-free survival [75]. However, CART33 treatment also lead to hematopoietic toxicity, peripheral blood cytopenia and decreased myeloid progenitor, which suggests toxicity due to permanently expressed CD33-CAR T-cells [75]. Therefore, an mRNA anti-CD33 CAR that is expressed for a short period of time was generated to decrease the potential toxicity [75].

High CD38 expression is a characteristic of MM [76]. An et al. (2018) produced a CD38-CAR using an anti-CD38 Nb [76]. The CD38-CAR T-cells specifically and efficiently lysed CD38+ MM cell lines and inhibited tumor growth in NOD/SCID mice (Table 3) [76]. Notably, the CD38-CAR T-cells showed low cytotoxicity in LP-1 cells (CD38 knocked out) and K562 cells (did not express CD38) as well as the CD38+ fractions of T-cells and B-cells [76].

Passive immunotherapy is divided into two categories: specific (an immune response to a certain tumor antigen) and non-specific (a general immune response) strategies [70]. It involves the initiation of host immune responses by the injection of externally produced components (Abs and cytokines) [70].

Cytokines (e.g., interferon gamma (IFN-γ) and ILs) are commonly used in cancer immunotherapy due to their wide range of anti-tumor activity, such as stimulating immune effector cells, increasing tumor cell recognition and activating cytotoxic cells [70,77]. However, certain cytokines, for instance, transforming growth factor beta (TGF-β) and IL-10, are immuno-suppressive; thus, studies are aimed at neutralizing these cytokines [70].

In advanced tumors, TGF-β overexpression was correlated with metastasis and a poor prognosis [78]. Previously, in preclinical models, TGF-β antagonism led to the prevention of metastasis without inducing a high level of toxicity [78]. Nam et al. (2008) reported that an anti-TGF-β Ab (1D11) suppressed metastasis and activated CD8-mediated anti-tumor immunity in a 4T1 breast cancer model [78]. Nbs can be generated against specific epitopes involved in the protein–receptor interaction, which may improve specificity and inhibitory function. Henry et al. (2016) produced sdAbs against a specific TGF-β3 epitope that is present at the site of the cytokine: receptor interaction [79]. The TGF-β3 sdAbs were shown to neutralize TGF-β3 (in vitro) and block the TGF-β3–receptor interaction (Table 3) [79]. In malignant conditions, these TGF-β3 sdAbs may be beneficial as a cancer immunotherapy [79].

Chemokines and chemokine receptors are overexpressed in a number of tumors and have roles in proliferation, metastasis and angiogenesis [80]. The chemokine receptor, CXCR7, has been shown to be overexpressed in various tumors (breast, lung, prostate, brain and kidney tumors) and may induce proliferation as well as angiogenesis [80]. In tumor models, conventional Abs have been used to inhibit CXCR7 and demonstrated promising effects [80]. Maussand et al. (2013) produced CXCR7 Nbs that inhibited tumor growth in a CXCR7-expressing head and neck cancer xenograft model (Table 3) [80]. Additionally, in these cancer cells, cell cycle progression was not inhibited, whereas CXCL1 (angiogenic chemokine) secretion was decreased by CXCR7 Nbs (in vitro) [80].

mAbs bind to specific overexpressed tumor antigens and stimulate the patient’s immune system, which results in inflammation and tumor cell destruction [70,81]. Under homeostatic conditions, immune checkpoints have essential roles in the maintenance of self-tolerance and the protection of host healthy tissues from damage that may occur during an immune response to a foreign substance (cancer antigens) [82]. The immune checkpoints include cytotoxic T lymphocyte-associated protein-4 (CTLA-4), programmed cell death-1 (PD-1), lymphocyte activated gene-3 (LAG-3), T-cell immunoglobulin and mucin-3 (TIM-3) and T-cell immunoglobulin and immunoreceptor tyrosine-based inhibitory motif domain (TIGIT). Malignant cells exploit the immune checkpoints in order to obtain immune resistance, escape destruction and survive [82]. Therefore, extensive research has been conducted on immune checkpoint blockades.

Upon stimulation through T-cell receptor (TCR) engagement, CTLA-4 moves to the cell surface and mediates the inhibition of T-cell proliferation and activation [17]. In animal models, a CTLA-4-blocking Ab was shown to strongly regress established tumors that lead to the clinical evaluation of immune checkpoint blockades [17]. In 2000, clinical trials involving ipilimumab and tremelimumab (anti-CTLA-4 Abs) began; however, strong tumor regressions were uncommon and certain toxicities (enterocolitis and inflammatory hepatitis) were experienced [17]. The use of immune suppressive drugs (corticosteroids) is able to treat these symptoms/toxicities without affecting anti-tumor activity [17]. The most evident clinical response to the CTLA-4 blockade (ipilimumab and tremelimumab) was seen in advanced metastatic melanoma patients [17,83,84]. More recently, in metastatic urothelial carcinoma patients, tremelimumab treatment showed clinical activity and anti-tumor responses [85]. Due to the minimal response rate and toxicities related to the CTLA-4 blockade, further research is required [17]. Wan et al. (2018) isolated an anti-CTLA-4 Nb 16 (Nb16) and demonstrated that Nb16 decreased melanoma growth and prolonged survival time in B16 melanoma-bearing C57BL/6 mice (Table 3) [86]. Another Nb against CTLA-4 (Nb16) was designed by Tang et al. (2019) [87]. In DC/HepG2-fused cells, Nb16 increased CD8+ T-cell proliferation and IFN-γ production, therefore resulting in elevated tumor cell killing (Table 3) [87].

Upon antigen presentation and TCR engagement, PD-1 expression is upregulated [88]; thereafter, PD-1 may bind to a PD-1 ligand (PD-L1 or PD-L2) [17,88,89]. The interaction between PD-1 and PD-L negatively regulates T-cell responses and decreases T-cell proliferation/differentiation, cytokine production (IFN-γ, tumor necrosis factor alpha (TNF-α) and IL-2) and cell survival [17,88,89,90]. In multiple cancer types, patient response rates have demonstrated the positive effect of the PD-1 pathway blockade [17]. Utilizing Abs to inhibit the PD-1–PD-L1 interaction leads to T-cell proliferation, penetration into the tumor and a cytotoxic T-cell response [17]. The first FDA-approved anti-PD-1 Abs were pembrolizumab and nivolumab for treating refractory melanoma (2014) and advanced NSCLC patients (2015) [17]. Nivolumab was the first Ab to demonstrate the anti-tumor activity of the PD-1 blockade [17]. In patients with melanoma, renal cell carcinoma (RCC) and NSCLC, treatment with nivolumab showed tumor responses [17]. Anti-tumor activity was accompanied with limited toxicity; however, rarely, pneumonitis may develop [17]. Thereafter, the anti-PD-L1 Abs atezolizumab and avelumab were approved for urothelial cancers (2016) and Merkel cell carcinoma (2017), respectively [17]. In metastatic urothelial carcinoma patients, avelumab treatment was linked to durable responses and a prolonged survival [91,92].

In NSCLC patients, progression-free survival was much longer in nivolumab- and ipilimumab-treated patients as compared to chemotherapy-treated patients [93]. Treatment with pembrolizumab and chemotherapy in previously untreated metastatic squamous NSCLC patients as well as treatment with atezolizumab and chemotherapy in extensive-stage SCLC resulted in a substantially longer survival and progression-free survival compared to chemotherapy alone [94,95]. Although PD-1 Abs have shown promising results, currently, the production of PD-1 and PD-L1 Nbs is of interest.

Xian et al. (2019) isolated a PD-1 Nb (Nb97) and generated an Nb97–Nb97-human serum albumin (HSA)-fused protein (MY2935) to extend half-life [96]. Although MY2935 (expressed in *P. pastoris*) and MY2626 (expressed in mammal cells, humanized Nb97-Fc) induced similar inhibitory effects on PD-1, MY2935 was more effective at blocking the PD-1–PD-L1 pathway (Table 3) than MY2626 [96]. Therefore, MY2935 stimulated the immune function to a greater extent than MY2626 [96]. More recently, an anti-PD-1 Nb (PD-1-Nb-B20) was selected by using a PD-1 peptide found on the PD-1 interface as the antigen [97]. In BxPC-3 cells, PD-1-Nb-B20 was shown to effectively block the binding of PD-1 and PD-L1 (Table 3) [97].

Additionally, Nbs have been produced against PD-L1 [98,99]. Zhang et al. (2017) produced an anti-PD-L1 Nb (KN035) and demonstrated that KN035-Fc blocked the PD-L1– PD-1 interaction, induced IFN-γ production and inhibited tumor growth (Table 3) [99]. Notably, KN035 demonstrated strong anti-tumor activity at comparable dosages of durvalumab [99].

LAG-3 inhibits TCR signaling by binding to MHC-II, thus preventing TCR and CD4 binding to the same MHC molecule [100]. Galectin-3 (Gal-3), liver sinusoidal endothelial cell lectin (LSECtin) and fibrinogen-like protein 1 (FGL1) are LAG-3 ligands that are frequently expressed in the tumor environment [101]. LAG-3 expression can inhibit TCR signaling, cell cycle progression and primary T-cell activation [100,102]. An increase in LAG-3 expression negatively modulates CD4+ T-cell activation and reduces CD8+ T-cell effector function [100]. The blockade of LAG-3 was shown to increase CD8+ T-cell effector function and stimulate higher IFN-γ production in self-tolerance models [103].

Yu et al. (2019) isolated an anti-LAG-3 Ab (LBL-007) and demonstrated that LBL-007 binds specifically to human LAG-3, induces IL-2 secretion, blocks the LAG-3–MHC class II interaction, the LAG-3–LSECtin interaction and LAG-3 signaling [104]. Additionally, LBL-007 treatment delayed tumor growth in mice transplanted with colorectal cancer cells [104].

Nbs targeting mouse LAG-3 were developed and validated as probes for SPECT imaging [101]. The ^99m^Tc-labeled-LAG-3 Nbs were shown to detect LAG-3 expression (Table 3) [101]. However, further research is required to investigate the anti-tumor potential of LAG-3 Nbs.

Gal-9, carcinoembryonic antigen cell adhesion molecule 1 (CEACAM-1), high-mobility group protein B1 (HMGB1) and phosphatidylserine (PS) are TIM-3 ligands [105]. The TIM-3–Gal-9 interaction mediates T-cell exhaustion, negatively regulates T-cell immunity, terminates Th1 immune responses and inhibits proliferation as well as effector cytokine production [105,106]. Moreover, innate immune cell functions (IL-12 secretion and toll-like receptor (TLR)-mediated activation) have been shown to be inhibited by the TIM-3–HMGB1 interaction [107].

In RCC patients, TIM-3 levels were elevated on T-cells (CD4+ and CD8+) and were linked to higher cancer stages [108]. Notably, the TIM-3 pathway blockade restored cell proliferation and elevated IFN-γ production in the TILs of RCC patients [108]. In CD8+ T-cells, TIM-3 was shown to be present at the immune synapse, disrupt stable synapse formation, interact with signaling molecules and contribute to the suppression of TCR signaling [109]. The formation of stable synapses was increased by the Tim-3 blockade [109].

Blocking the TIM-3–ligand interaction would restore effector functions and the anti-tumor activity of exhausted T-cells [110]. The development of a TIM-3 blocking agent could be a beneficial immunotherapy for cancer [106]. Previously, an anti-TIM-3 Ab (M6903) was shown to effectively bind to TIM-3, block TIM-3 binding to its ligands (PS, CEACAM1 and Gal-9) and enhance T-cell activation [111]. The combination of M6903 and bintrafusp alfa (a protein that blocks the TGF-β and PD-L1 pathways) enhanced T-cell activation and anti-tumor efficacy [111].

Homayouni et al. (2016) produced an anti-human TIM-3 Nb (CD366) and demonstrated its specific reactivity and high binding capacity to TIM-3 [106]. Notably, the binding capacity of the anti-TIM-3 Nb was similar to a commercial Ab [106]. Additionally, in HL-60 cells, the anti-TIM-3 Nb exerted a high anti-proliferative effect (Table 3), which was comparable with an anti-TIM-3 Ab [106].

Upon activation, TIGIT expression was shown to be increased on T-cells [112]. The TIGIT immune checkpoint has three ligands, namely CD155, CD112 and CD113 [112]. However, TIGIT binds with higher affinity to CD155 (main TIGIT ligand) [112]. In many human malignancies, the overexpression of CD155 and CD112 has been evident [112]. Moreover, oncogene and cytokine (IFN-γ) expression have been shown to increase CD155 and CD112 on tumor cells [112]. The TIGIT–CD155 interaction suppresses IFN-γ production [113]. Additionally, it increases IL-10 production, which plays a role in T-cell exhaustion and immunosuppression [113]. In cytokine-induced killer cells, an anti-TIGIT Ab elevated proliferation, cytokine production and the cytotoxic targeting of tumor cells expressing CD155 [113].

Immune checkpoint blockades allow for the continuous anti-tumor activity of T-cells, which suggests long-lasting responses against cancer [17]. However, acquired resistance is a problem with checkpoint blockade therapy [17]. The immune checkpoint receptors can be expressed simultaneously. Therefore, studies have examined the effect of blocking more than one immune checkpoint.

Previously, in patients with advanced melanoma, RCC and NSCLC, the inhibition of both the PD-1 and CTLA-4 pathways elevated survival and objective response rates [114,115]. Additionally, in ipilimumab (CTLA-4 antibody)-treated metastatic melanoma patients, there was a greater number of cells expressing intracellular CTLA-4, LAG-3 and TIM-3 [116]. Thus, the effectiveness of targeting LAG-3, PD-1 and CTLA- 4 is under clinical investigation [100].

Studies have reported that PD-1 and LAG-3 are co-expressed on CD4+, CD8+ and tumor-infiltrating T-cells [117,118,119]. Additionally, in cancer models, the synergy between LAG-3 and PD-1 has been noted [100]. Most established tumors tend to be resistant to a single agent treatment [100]. However, these tumors are effectively eliminated by simultaneously blocking LAG-3 and PD-1 [100].

In human NSCLC, the expression of LAG-3 on TILs was significantly associated with PD-1 expression on TILs as well as PD-L1 expression on tumors [118]. Additionally, in NSCLC patients, a low expression of LAG-3 and PD-L1 has shown a favorable prognosis as compared to increased LAG-3 and PD-L1 expression [118]. In patients treated with anti-PD-1 therapy, LAG-3 expression is linked to a shorter progression-free survival [120]. Notably, the anti-LAG-3 Ab and anti-PD-1 Ab combination treatment more effectively delayed tumor growth than the single treatments (LBL-007 only or anti-PD-1 Ab only) [104].

In in vitro and in vivo models, the expression of TIM-3 and PD-1 was correlated [105]. In gastric cancer patients, PD-1- and TIM-3-positive CD8+ T-cells were increased, and PD-1+ cells were correlated with TIM-3+ cells [121]. Moreover, the level of IFN-γ produced by PD-1- and TIM-3-positive T-cells was substantially lower than that of PD-1- and TIM-3-negative cells as well as PD-1-positive and TIM-3-negative cells [121]. A combination of TIM-3 and PD-1 inhibitors could prevent tumor progression and improve anti-tumor activity [105,110]. The blockade of both PD-1–PD-L1 and TIM-3–Gal-9 interactions could prevent T-cell exhaustion in advanced acute myelogenous leukemia (AML) as well as reversed T-cell dysfunction and exhaustion in colorectal cancer [122,123].

A previous study has reported that in in vivo models of lung cancer, there is an increase in alternative immune checkpoints following anti-PD-1 therapy [124]. Thus, the adaptive resistance to the PD-1 pathway blockade may be due to the increased expression of other immune checkpoints [124]. In hepatocellular carcinoma (HCC) tissues, the expression of PD-1, TIM-3 and LAG-3 was increased on T-cells [125]. Abs against PD-L1, TIM-3 or LAG-3 increased T-cell proliferation and cytokine production and restored the T-cell responses of HCC-derived T-cells [125]. Therefore, the combined inhibition of PD-1, TIM-3 or LAG-3 demonstrated a synergistic function [125].

In cancer patients, TIGIT expression is often correlated with the increased expression of inhibitory receptors (PD-1, LAG-3 and TIM-3) on CD8+ TILs [112,126]. TIGIT and PD-1 have been shown to be co-expressed in advanced melanoma, NSCLC and colon cancer samples [126]. In cells isolated from metastatic melanoma patients, blocking both TIGIT and PD-1 was shown to increase proliferation and cytokine production [126]. Recently, Ma et al. (2020) produced a multivalent bispecific Ab (BsAb) consisting of a tetravalent anti-PD-L1 Fc-fusion Nb and tetravalent anti-TIGIT Nb [127]. The parental anti-PD-L1 Nb demonstrated high specificity and affinity for PD-L1 as well as increased T-cell activity (in vitro) and anti-tumor activity (in vivo) (Table 3) [127]. The parental anti-TIGIT Nb also showed high specificity and affinity for TIGIT as well as enhanced T-cell activity (Table 3). In comparison to the parental Nbs, BsAb was shown to co-target PD-L1 and TIGIT and highly block the receptor–ligand interaction as well as synergistically increase T-cell activity (in vitro) (Table 3) [127].

Taken together, the blockade of more than one immune checkpoint may prove to be a very beneficial cancer treatment [100].

### 4.8. Anti-Cancer Treatments

Over the years, Abs and Nbs have been widely researched to enhance oncology practices, such as imaging, radiotherapy and drug delivery, in which Abs/Nbs are conjugated to other components. However, Abs and Nbs tend to be initially developed to demonstrate anti-cancer potential, for example, EGRF, hepatocyte growth factor (HGF) and death receptor 5 (DR5).

In EGFR-overexpressing MDA-MB-468 breast cancer cells, the combined use of cetuximab and matuzumab (anti-EGFR mAbs) decreased growth and survival to a greater extent than the use of a single Ab treatment [22]. Different Abs may bind to different epitopes of a target [21]. Roovers et al. (2011) produced a single biparatopic anti-EFGR Nb (CONAN-1) by combining Nbs that possess specificities that are similar to cetuximab and matuzumab [21]. CONAN-1 demonstrated an enhanced affinity for EFGR, blocked EFGR activation and inhibited cell proliferation (Table 3) [21]. Additionally, in athymic mice bearing A431 xenografts, CONAN-1 was shown to inhibit tumor growth similar to cetuximab and to a greater extent than bivalent monospecific Nbs [21]. Anti-EGFR Nbs (ENb) have also been produced and secreted from stem cells for glioblastoma multiforme (GBM) therapy [128]. In GBM cells lines, van der Water et al. (2012) demonstrated that ENbs inhibited EGFR signaling, which led to decreased growth and invasiveness (Table 3) [128].

Solid tumors have been shown to overexpress EGFR, and a poor prognosis of patients has been correlated with increased EGFR expression [129]. In the treatment of solid tumors, EGFR Abs have been promising; however, the combination of EGFR Abs and conventional therapies (chemo and radio) is more beneficial [129]. Roovers et al. (2007) isolated anti-EGFR Nbs that inhibited EGF binding and blocked EGF-mediated signaling and cell proliferation [129]. Notably, the anti-EGFR Nbs were shown to delay A431-derived solid tumor outgrowth (in vivo) (Table 3) [129].

HGF is a ligand for the c-Met receptor [12]. In cancer patients, HGF and c-Met overexpression is related to elevated tumor aggressiveness and a poor prognostic outcome [12]. Therefore, inhibiting HGF expression may be a beneficial strategy to decrease c-Met receptor activation [12]. For decades, pharmaceutical companies have been developing mAbs that antagonize c-Met activation, and three anti-HGF mAbs (AMG102 (rilotumumab), AV-299 and TAK-701) have been under clinical development [130]. Notably, in patients with recurrent glioblastoma, rilotumumab was not related to significant anti-tumor activity [131].

Two HGF Nbs (1E2 and 6E10) were shown to inhibit HGF–c-Met receptor binding [12]. The HGF Nbs were linked to albumin (Alb8) to extend serum half-life and radiolabeled with ^89^Zr (^89^Zr-1E2-Alb8 and ^89^Zr-6E10-Alb8) to allow an in vivo assessment of biodistribution [12]. In U87MG xenografts, ^89^Zr-1E2-Alb8 and ^89^Zr-6E10-Alb8 were shown to selectively target tumors (Table 3) [12]. Additionally, in U87 MG tumor-bearing nude mice, HGF Nbs inhibited tumor growth, and cures were observed in 50–67% of mice, indicating therapeutic potential [12]. In lung tumors, increased c-Met gene amplification may contribute to the resistance to EGFR inhibitors [12]. NSCLC tumors with an EGFR mutation eventually become resistant to EGFR tyrosine kinase inhibitors (TKI) [132]. In these NSCLC cells, HGF induces resistance to EGFR-TKI similar to gefitinib [132]. Okamoto et al. (2010) demonstrated that HGF activates MET, which confers gefitinib resistance [132]. In gefitinib-resistant NSCLC tumors, an anti-HGF mAb (TAK-701) was shown to restore gefitinib sensitivity [132]. Thus, constructing an Nb that includes an anti-HGF Nb and anti-EGFR Nb may enhance therapeutic potential by simultaneously inhibiting EGFR and c-Met–HGF signaling [12].

DR5 is involved in the activation of extrinsic apoptosis [133]. In preclinical models, DR5 agonists have showed promising anti-tumor activity; however, in cancer patients, its efficacy was insufficient [133]. Huet et al. (2014) produced multivalent DR5 agonist Nbs that increased tumor cell killing by caspase induction [133]. In comparison to the DR5 agonist Ab, the multivalent DR5 Nb showed enhanced anti-tumor activity (in vivo) (Table 3) [133].

## 5. Conclusions

Cancer is a devastating disease and a leading cause of mortality worldwide. For decades, Abs have been developed and widely used in oncology practices. However, there are disadvantages with the use of Abs in cancer imaging and therapy. In comparison to Abs, Nbs have several advantages, which may be beneficial [7].

In cancer practices, the literature indicates that the usage of Nbs can be equally or more effective than Abs while producing a lower level of toxicity and adverse effects. Nbs have shown the potential to improve cancer visualization, drug delivery and therapies (radiotherapy, chemotherapy and surgery). Additionally, Nbs can be developed specifically as a cancer treatment. However, further research into the development and utilization of Nbs is required in order to assess their safety and efficacy for patient administration.

## Figures and Tables

**Table 1:** A comparison between conventional antibodies and nanobodies.

Antibody	Nanobody
High immunogenicity levels	Low immunogenicity levels
Large size (150 kDa)	Small size (12–14 kDa)
Long half-life	Short half-life
Limited tumor penetration	Deep tumor penetration
CDR3 length is average	CDR3 length is long
Antigen-interacting surface is flat with limited flexibility	Finger-like structure for antigen interaction
Fragile	High stability
Prone to degradation, changes in temperature and pH	Resistant to degradation. Tolerant to temperature, pressure and pH changes
Expensive large-scale production	Inexpensive large-scale production
Mammalian expression	Microbial expression
Complex structure and post-translational modifications	Simple structure and lack of post-translational modifications
	Easily formatted into various constructs
	Engineered to suit treatment

**Table 2:** A comparison between antibodies and nanobodies in molecular imaging, diagnosis and surgery.

Antibody	Nanobody
Slow and non-specific tumor accumulation	Rapid and specific tumor accumulation
Slow imaging time (1–6 days)	Rapid imaging time (1–3 h)
Increased background-to-tumor ratio	High tumor-to-background ratio
Slow clearance	Rapid clearance
Less accurate visualization of tumor, metastasized lesions and tumor boarders	More accurate visualization of tumor, metastasized lesions and tumor boarders

**Table 3:** The effects of nanobodies in preclinical and clinical studies.

**Preclinical Studies**		
**Nanobody and Conjugates**	**Effects**	**Reference**
^99m^Tc-2Rs15d ^99m^Tc-EGFR	High tumor and renal uptake. Rapid blood clearance	[25,26]
^99m^Tc-PSMA30	High tumor uptake. High tumor-to-normal organ ratios	[27]
^99m^Tc-anti-MMR	Target and image TAM subpopulations	[14]
^99^mTc-R3B23	Image and monitor progression of the disease	[31]
^68^Ga-EGFR	High tumor uptake and high tumor-to-normal tissue ratios	[32]
^68^Ga-NOTA-2Rs15d	Fast and specific uptake. High tumor-to-blood ratios. High-specific contrast images. No observed toxicity	[29]
μB-cAbVCAM1-5	Imaging of tumors	[33]
anti-PSMA nBs	Target PSMA+ cells to image prostate cancer	[35]
anti-CAIX Nb-IRDye800CW	Imaging of pre-invasive breast cancer	[36]
7D12-IRDye800CW	High tumor uptake. Rapid and clear imaging of orthotopic tongue tumors and cervical lymph node metastases	[37]
11A4-IRDye800CW	Increased tumor accumulation and tumor-to-background ratios. Used in image-guided surgery	[42]
^177^Lu-DTPA-sdAb-9079	Effective treatment. Delivers low radiation levels to healthy organs	[46]
^213^Bi-2Rs15d	High tumor uptake, low healthy tissue uptake and increased median survival	[49]
^177^Lu-DTPA-2Rs15d	High dose delivered to the tumor and kidneys. Tumor growth blockade and a substantial event-free survival	[50]
anti-VEGFR2 Nb-PE38	Inhibit proliferation of VEGFR2-expressing cells	[54]
anti-EGFR Nb-cucurmosin	Inhibit cell viability of EGFR-expressing cell lines and induce apoptosis.	[57]
Bispecific anti-EGFR-Nb-cucurmosin	Selectively kills cancer cells through apoptosis	[58]
anti-HER-2-PE24X7	High selectivity and cytotoxicity. Effective tumor growth inhibition	[59]
EGa1-L	Decreased EGFR levels, EGFR sequestration, receptor degradation and inhibition of tumor cell proliferation	[60]
AG538-loaded EGa1-liposomes	AG538 delivery, EGFR blockade, IGF-1R stimulation and inhibition of tumor cell proliferation	[61]
Rhodamine-labeled EGa1-micelles	High binding and uptake through EGF receptor interaction. Tumor growth inhibition	[62,63]
anti-Met NANAPs	Binding, uptake and lysosomal degradation. Downregulation of Met protein	[64]
Ega1-coated NANAPs	Internalized, digested, kinase inhibitor release and decreased cancer cell proliferation	[65]
doxorubicin-EGa1-polymeric-micelles	Effective inhibition of tumor growth	[63]
NB7-doxorubicin	Accumulates in PSMA+ tumors. Doxorubicin induced cytotoxic activity. Tumor growth inhibition at a low dosage	[30]
CD38-CAR T-cells	Specific and efficient lyses of CD38+ MM cell lines and inhibition of tumor growth	[76]
TGF-β3 sdAbs	Neutralize TGF-β3 and block the TGF-β3–receptor interaction	[79]
CXCR7 Nbs	Inhibition of tumor growth	[80]
Nb16 and Nb16	Decreases melanoma growth and prolongs survival time. Increases T-cell proliferation and IFN-γ production. Increases tumor cell killing	[86,87]
MY2935	Effective blockade of the PD-1–PD-L1 pathway	[96]
PD-1-Nb-B20	Effectively block the binding of PD-1 and PD-L1	[97]
KN035	Blockade of the PD-L1–PD-1 interaction, induction of IFN-γ production and inhibition of tumor growth	[99]
^99m^Tc-LAG-3 Nbs	Detect LAG-3 expression	[101]
anti-human TIM-3 Nb	Specific reactivity and high binding capacity. High anti-proliferative effect	[106]
anti-PD-L1 Nb	High specificity and affinity for PD-L1. Increased T-cell activity and anti-tumor activity	[127]
anti-TIGIT Nb	High specificity and affinity for TIGIT. Enhanced T-cell activity	[127]
BsAb	Co-target PD-L1 and TIGIT, highly block the receptor–ligand interaction and increase T-cell activity	[127]
CONAN-1	High affinity for EFGR, blocks EFGR activation, inhibition of cell proliferation and tumor growth	[21]
ENb	Inhibition of EGFR signaling. Decreases growth and invasiveness	[128]
anti-EGFR Nbs	Inhibition of EGF binding, blockade of EGF-mediated signaling and cell proliferation. Delays solid tumor outgrowth	[129]
^89^Zr-1E2-Alb8 ^89^Zr-6E10-Alb8	Selectively target tumors and inhibits tumor growth	[12]
DR5 Nb	Increases tumor cell killing by caspase induction. Increases anti-tumor activity	[133]
**Clinical Studies**		
**Nanobody and Conjugates**	**Effects**	**Reference**
^99m^Tc-NM-01	Imaging of PD-L1-expressing cells. No adverse effects. Monitors PD-L1 expression. Useful in diagnosing and staging of patients	[39]

## Data Availability

Not applicable.

## Abbreviations

Mil, million; Igs, immunoglobulins; Abs, antibodies; VH, variable heavy; VL, variable light; mAbs, monoclonal Abs; Fab, synthetic antigen-binding fragment; Fv, variable fragment; scFv, single-chain Fv; CDR, complementarity-determining regions; Nbs, nanobodies; ABF, antigen-binding fragment; SPECT, single-photon emission computed tomography; PET, positron emission tomography; CT, *computed tomography;* ^99^mTc, 99-mTechnetium; ^177^Lu, 177-lutetium; ^123^I, 123-iodine; ^11^I, 11-iodine; ^68^Ga, 68-gallium; ^124^I, 124-iodine; ^89^Zr, zirconium-89; ^18^F, fluor-18; PD-1, programmed cell death-1; PD-L1, PD-1 ligand; ^111^In, 111-indium; p.i., post injection; ^64^Cu, 64-copper; EGFR, epidermal growth factor receptor; ESCC, esophageal squamous cell carcinoma; h, hours; HER-2, human epidermal growth factor receptor 2; PSMA, prostate-specific membrane antigen; MMR, macrophage mannose receptor; TAM, tumor-associated macrophages; MM, multiple myeloma; M protein, monoclonal protein; µBs, microbubbles; VCAM-1, vascular cell adhesion molecule-1; nBs, nanobubbles; NIR, near-infrared dyes; CAIX, carbonic anhydrase IX; NSCLC, non-small cell lung cancer; TRNT, targeted radionuclide therapy; ^131^I, 131-iodine; ^89^Sr, 89-strontium; LET, linear energy transfer; ^90^Y, yttrium-90; ^212^Bi, 212-bismuth; ^225^Ac, 225-actinium; ^223^Ra, 223-radium; FDA, Food and Drug administration; RIT, radioimmunotherapy; BRT, blood residence time; sdAb, single-domain Ab fragment; ^213^Bi, 213-bismuth; ABD, albumin-binding domain; VEGFR2, vascular endothelial growth factor receptor 2; NANAPs, albumin-based nanoparticles; IGF-1R, insulin-like growth factor 1 receptor; Tra, trastuzumab; Ps, polymersomes; EPI·HCL, epirubicin hydrochloride; ACT, adoptive cell transfer; TILs, tumor-infiltrating lymphocytes; CARs, chimeric antigen receptors; IL, interleukin; AML, acute myeloid leukemia; IFN-γ, interferon gamma; TGF-β, transforming growth factor β; CTLA-4, cytotoxic T lymphocyte-associated protein-4; PD-1, programmed cell death-1; LAG-3, lymphocyte-activated gene-3; TIM-3, T-cell immunoglobulin and mucin-3; TIGIT, T-cell immunoglobulin and immunoreceptor tyrosine-based inhibitory motif domain; TCR, T-cell receptor; TNF-α, tumor necrosis factor alpha; HSA, human serum albumin; Gal-3, galectin-3; LSECtin, liver sinusoidal endothelial cell lectin; FGL1, fibrinogen-like protein 1; CEACAM-1, carcinoembryonic antigen cell adhesion molecule; HMGB1, high-mobility group protein B1; PS, phosphatidylserine; TLR, toll-like receptor; RCC, renal cell carcinoma; HCC, hepatocellular carcinoma.

## References

[1] WHO (2020). WHO Report on Cancer: Setting Priorities, Investing Wisely and Providing Care for All.

[2] Lin W.W., Karin M. (2007). A cytokine-mediated link between innate immunity, inflammation, and cancer. J. Clin. Investig..

[3] Bray F., Ferlay J., Soerjomataram I., Siegel R.L., Torre L.A., Jemal A. (2018). Global Cancer Statistics 2018: GLOBOCAN Estimates of Incidence and Mortality Worldwide for 36 Cancers in 185 Countries. CA Cancer J. Clin..

[4] Mattiuzzi C., Lippi G. (2019). Current Cancer Epidemiology. J. Epidemiol. Glob. Health.

[5] WHO (2016). Global Health Estimates 2016: Disease Burden by Cause, Age, Sex, by Country and by Region, 2000–2016.

[6] WHO International Agency for Research on Cancer. Global Cancer Observatory—Cancer Fact Sheets. http://gco.iarc.fr/today/fact-sheets-cancers.

[7] Jovčevska I., Muyldermans S. (2020). The Therapeutic Potential of Nanobodies. BioDrugs.

[8] Oliveira S., Heukers R., Sornkom J., Kok R.J., van Bergen en Henegouwen P.M.P. (2013). Targeting tumors with nanobodies for cancer imaging and therapy. J. Control. Release.

[9] Siontorou C.G. (2013). Nanobodies as novel agents for disease diagnosis and therapy. Int. J. Nanomed..

[10] D’Huyvetter M., Xavier C., Caveliers V., Lahoutte T., Muyldermans S., Devoogdt N. (2014). Radiolabeled nanobodies as theranostic tools in targeted radionuclide therapy of cancer. Expert Opin. Drug Deliv..

[11] Van Audenhove I., Gettemans J. (2016). Nanobodies as versatile tools to understand, diagnose, visualize and treat cancer. EBioMedicine.

[12] Vosjan M.J.W.D., Vercammen J., Kolkman J.A., Stigter-van Walsum M., Revets H., van Dongen G.A.M.S. (2012). Nanobodies Targeting the Hepatocyte Growth Factor: Potential New Drugs for Molecular Cancer Therapy. Mol. Cancer Ther..

[13] Tijink B.M., Laeremans T., Budde M., Stigter-van Walsum M., Dreier T., de Haard H.J., Leemans C.R., van Dongen G.A.M.S. (2008). Improved tumor targeting of anti-epidermal growth factor receptor Nanobodies through albumin binding: Taking advantage of modular Nanobody technology. Mol. Cancer Ther..

[14] Movahedi K., Schoonooghe S., Laoui D., Houbracken I., Waelput W., Breckpot K., Bouwens L., Lahoutte T., De Baetselier P., Raes G. (2012). Nanobody-based targeting of the macrophage mannose receptor for effective in vivo imaging of tumor-associated macrophages. Cancer Res..

[15] Arruebo M., Vilaboa N., Sáez-Gutierrez B., Lambea J., Tres A., Valladares M., González-Fernández A. (2011). Assessment of the Evolution of Cancer Treatment Therapies. Cancers.

[16] Symonds R.P., Foweraker K. (2006). Principles of chemotherapy and radiotherapy. Curr. Obstet. Gynaecol..

[17] Ribas A., Wolchok J.D. (2018). Cancer immunotherapy using checkpoint blockade. Science.

[18] Heskamp S., Hobo W., Molkenboer-Kuenen J.D.M., Olive D., Oyen W.J.G., Dolstra H., Boerman O.C. (2015). Noninvasive Imaging of Tumor PD-L1 Expression Using Radiolabeled Anti-PD-L1 Antibodies. Cancer Res..

[19] Chatterjee S., Lesniak W.G., Gabrielson M., Lisok A., Wharram B., Sysa-Shah P., Azad B.B., Pomper M.G., Nimmagadda S. (2016). A humanized antibody for imaging immune checkpoint ligand PD-L1 expression in tumors. Oncotarget.

[20] Kobayashi K., Sasaki T., Takenaka F., Yakushiji H., Fujii Y., Kishi Y., Kita S., Shen L., Kumon H., Matsuura E. (2015). A novel PET imaging using ⁶⁴Cu-labeled monoclonal antibody against mesothelin commonly expressed on cancer cells. J. Immunol. Res. 2015..

[21] Roovers R.C., Vosjan M.J.W.D., Laeremans T., el Khoulati R., de Bruin R.C.G., Ferguson K.M., Verkleij A.J., van Dongen G.A.M.S., van Bergen en Henegouwen P.M.P. (2011). A biparatopic anti-EGFR nanobody efficiently inhibits solid tumour growth. Int. J. Cancer.

[22] Kamat V., Donaldson J.M., Kari C., Quadros M.R.D., Lelkes P.I., Chaiken I., Cocklin S., Williams J.C., Papazoglou E., Rodeck U. (2008). Enhanced EGFR inhibition and distinct epitope recognition by EGFR antagonistic mAbs C225 and 425. Cancer Biol. Ther..

[23] Menke-van der Houven C.W., Gootjes E.C., Huisman M.C., Vugts D.J., Roth C., Luik A.M., Mulder E.R., Schuit R.C., Boellaard R., Hoekstra O.S. (2015). 89Zr-cetuximab PET imaging in patients with advanced colorectal cancer. Oncotarget.

[24] Song I.H., Lee T.S., Park Y.S., Lee J.S., Lee B.C., Moon B.S., An G.I., Lee H.W., Kim K.I., Lee Y.J. (2016). Immuno-PET Imaging and Radioimmunotherapy of 64Cu-/177Lu-Labeled Anti-EGFR Antibody in Esophageal Squamous Cell Carcinoma Model. J. Nucl. Med..

[25] Gainkam L.O.T., Huang L., Caveliers V., Keyaerts M., Hernot S., Vaneycken I., Vanhove C., Revets H., De Baetselier P., Lahoutte T. (2008). Comparison of the biodistribution and tumor targeting of two 99mTc-labeled anti-EGFR nanobodies in mice, using pinhole SPECT/micro-CT. J. Nucl. Med..

[26] Vaneycken I., Devoogdt N., Van Gassen N., Vincke C., Xavier C., Wernery U., Muyldermans S., Lahoutte T., Caveliers V. (2011). Preclinical screening of anti-HER2 nanobodies for molecular imaging of breast cancer. FASEB J..

[27] Evazalipour M., D’Huyvetter M., Tehrani B.S., Abolhassani M., Omidfar K., Abdoli S., Arezumand R., Morovvati H., Lahoutte T., Muyldermans S. (2014). Generation and characterization of nanobodies targeting PSMA for molecular imaging of prostate cancer. Contrast Media Mol. Imaging.

[28] Krop I.E., Beeram M., Modi S., Jones S.F., Holden S.N., Yu W., Girish S., Tibbitts J., Yi J.H., Sliwkowski M.X. (2010). Phase I study of trastuzumab-DM1, an HER2 antibody-drug conjugate, given every 3 weeks to patients with HER2-positive metastatic breast cancer. J. Clin. Oncol..

[29] Xavier C., Vaneycken I., D’huyvetter M., Heemskerk J., Keyaerts M., Vincke C., Devoogdt N., Muyldermans S., Lahoutte T., Caveliers V. (2013). Synthesis, preclinical validation, dosimetry, and toxicity of 68Ga-NOTA-anti-HER2 Nanobodies for iPET imaging of HER2 receptor expression in cancer. J. Nucl. Med..

[30] Rosenfeld L., Sananes A., Zur Y., Cohen S., Dhara K., Gelkop S., Ben Zeev E., Shahar A., Lobel L., Akabayov B. (2020). Nanobodies Targeting Prostate-Specific Membrane Antigen for the Imaging and Therapy of Prostate Cancer. J. Med. Chem..

[31] Lemaire M., D’Huyvetter M., Lahoutte T., Van Valckenborgh E., Menu E., De Bruyne E., Kronenberger P., Wernery U., Muyldermans S., Devoogdt N. (2014). Imaging and radioimmunotherapy of multiple myeloma with anti-idiotypic Nanobodies. Leukemia.

[32] Vosjan M.J.W.D., Perk L.R., Roovers R.C., Visser G.W.M., Stigter-van Walsum M., van Bergen En Henegouwen P.M.P., van Dongen G.A.M.S. (2011). Facile labelling of an anti-epidermal growth factor receptor Nanobody with 68Ga via a novel bifunctional desferal chelate for immuno-PET. Eur. J. Nucl. Med. Mol. Imaging.

[33] Hernot S., Unnikrishnan S., Du Z., Shevchenko T., Cosyns B., Broisat A., Toczek J., Caveliers V., Muyldermans S., Lahoutte T. (2012). Nanobody-coupled microbubbles as novel molecular tracer. J. Control. Release.

[34] Kong D.H., Kim Y.K., Kim M.R., Jang J.H., Lee S. (2018). Emerging Roles of Vascular Cell Adhesion Molecule-1 (VCAM-1) in Immunological Disorders and Cancer. Int. J. Mol. Sci..

[35] Fan X., Wang L., Guo Y., Tu Z., Li L., Tong H., Xu Y., Li R., Fang K. (2015). Ultrasonic Nanobubbles Carrying Anti-PSMA Nanobody: Construction and Application in Prostate Cancer-Targeted Imaging. PLoS ONE.

[36] van Brussel A.S.A., Adams A., Oliveira S., Dorresteijn B., El Khattabi M., Vermeulen J.F., Van Der Wall E., Mali W.P., Derksen P.W.B., Van Diest P.J. (2015). Hypoxia-targeting fluorescent nanobodies for optical molecular imaging of pre-invasive breast cancer. Mol. Imaging Biol..

[37] van Driel P.B.A.A., van der Vorst J.R., Verbeek F.P.R., Oliveira S., Snoeks T.J.A., Keereweer S., Chan B., Boonstra M.C., Frangioni J.V., Van Bergen en Henegouwen P.M.P. (2014). Intraoperative fluorescence delineation of head and neck cancer with a fluorescent anti-epidermal growth factor receptor nanobody. Int. J. Cancer.

[38] Oliveira S., van Dongen G.A.M.S., Stigter-van Walsum M., Roovers R.C., Stam J.C., Mali W., van Diest P.J., van Bergen en Henegouwen P.M.P. (2012). Rapid visualization of human tumor xenografts through optical imaging with a near-infrared fluorescent anti-epidermal growth factor receptor nanobody. Mol. Imaging.

[39] Xing Y., Chand G., Liu C., Cook G.J.R., O’Doherty J., Zhao L., Wong N.C.L., Meszaros L.K., Ting H.H., Zhao J. (2019). Early Phase I Study of a 99mTc-Labeled Anti-Programmed Death Ligand-1 (PD-L1) Single-Domain Antibody in SPECT/CT Assessment of PD-L1 Expression in Non-Small Cell Lung Cancer. J. Nucl. Med..

[40] Sullivan R., Alatise O.I., Anderson B.O., Audisio R., Autier P., Aggarwal A., Balch C., Brennan M.F., Dare A., D’Cruz A. (2015). Global cancer surgery: Delivering safe, affordable, and timely cancer surgery. Lancet Oncol..

[41] Deken M.M., Bos D.L., Tummers W.S.F.J., March T.L., van de Velde C.J.H., Rijpkema M., Vahrmeijer A.L. (2019). Multimodal image-guided surgery of HER2-positive breast cancer using [111In]In-DTPA-trastuzumab-IRDye800CW in an orthotopic breast tumor model. EJNMMI Res..

[42] Kijanka M., Warnders F.J., El Khattabi M., Lub-De Hooge M., Van Dam G.M., Ntziachristos V., De Vries L., Oliveira S., van Bergen en Henegouwen P.M.P. (2013). Rapid optical imaging of human breast tumour xenografts using anti-HER2 VHHs site-directly conjugated to IRDye 800CW for image-guided surgery. Eur. J. Nucl. Med. Mol. Imaging.

[43] Baskar R., Lee K.A., Yeo R., Yeoh K.W. (2012). Cancer and Radiation Therapy: Current Advances and Future Directions. Int. J. Med. Sci..

[44] Zhang Q.Y., Wang F.X., Jia K.K., Kong L.D. (2018). Natural Product Interventions for Chemotherapy and Radiotherapy-Induced Side Effects. Front. Pharmacol..

[45] Rasaneh S., Rajabi H., Hossein Babaei M., Johari Daha F. (2010). Toxicity of trastuzumab labeled 177Lu on MCF7 and SKBr3 cell lines. Appl. Radiat. Isot..

[46] Krasniqi A., D’Huyvetter M., Xavier C., Van der Jeught K., Muyldermans S., Van Der Heyden J., Lahoutte T., Tavernier J., Devoogdt N. (2017). Theranostic Radiolabeled Anti-CD20 sdAb for Targeted Radionuclide Therapy of Non-Hodgkin Lymphoma. Mol. Cancer Ther..

[47] Liersch T., Meller J., Kulle B., Behr T.M., Markus P., Langer C., Ghadimi B.M., Wegener W.A., Kovacs J., Horak I.D. (2005). Phase II Trial of Carcinoembryonic Antigen Radioimmunotherapy with 131I-Labetuzumab after Salvage Resection of Colorectal Metastases in the Liver: Five-Year Safety and Efficacy Results. J. Clin. Oncol..

[48] Reardon D.A., Akabani G., Coleman R.E., Friedman A.H., Friedman H.S., Herndon J.E., McLendon R.E., Pegram C.N., Provenzale J.M., Quinn J.A. (2006). Salvage radioimmunotherapy with murine iodine-131-labeled antitenascin monoclonal antibody 81C6 for patients with recurrent primary and metastatic malignant brain tumors: Phase II study results. J. Clin. Oncol..

[49] Dekempeneer Y., Caveliers V., Ooms M., Maertens D., Gysemans M., Lahoutte T., Xavier C., Lecocq Q., Maes K., Covens P. (2020). Therapeutic Efficacy of 213Bi-labeled sdAbs in a Preclinical Model of Ovarian Cancer. Mol. Pharm..

[50] D’Huyvetter M., Vincke C., Xavier C., Aerts A., Impens N., Baatout S., De Raeve H., Muyldermans S., Caveliers V., Devoogdt N. (2014). Targeted radionuclide therapy with A 177Lu-labeled anti-HER2 nanobody. Theranostics.

[51] Gainkam L.O.T., Caveliers V., Devoogdt N., Vanhove C., Xavier C., Boerman O., Muyldermans S., Bossuyt A., Lahoutte T. (2011). Localization, mechanism and reduction of renal retention of technetium-99m labeled epidermal growth factor receptor-specific nanobody in mice. Contrast Media Mol. Imaging.

[52] Lewis Phillips G.D., Li G., Dugger D.L., Crocker L.M., Parsons K.L., Mai E., Blättler W.A., Lambert J.M., Chari R.V.J., Lutz R.J. (2008). Targeting HER2-positive breast cancer with trastuzumab-DM1, an antibody-cytotoxic drug conjugate. Cancer Res..

[53] Ogitani Y., Aida T., Hagihara K., Yamaguchi J., Ishii C., Harada N., Soma M., Okamoto H., Oitate M., Arakawa S. (2016). DS-8201a, A Novel HER2-Targeting ADC with a Novel DNA Topoisomerase I Inhibitor, Demonstrates a Promising Antitumor Efficacy with Differentiation from T-DM1. Clin. Cancer Res..

[54] Behdani M., Zeinali S., Karimipour M., Khanahmad H., Schoonooghe S., Aslemarz A., Seyed N., Moazami-Godarzi R., Baniahmad F., Habibi-Anbouhi M. (2013). Development of VEGFR2-specific Nanobody Pseudomonas exotoxin A conjugated to provide efficient inhibition of tumor cell growth. N. Biotechnol..

[55] Kreitman R.J., Pastan I. (2011). Antibody fusion proteins: Anti-CD22 recombinant immunotoxin moxetumomab pasudotox. Clin. Cancer Res..

[56] Kreitman R.J., Tallman M.S., Robak T., Coutre S., Wilson W.H., Stetler-Stevenson M., Fitzgerald D.J., Lechleider R., Pastan I. (2012). Phase I trial of anti-CD22 recombinant immunotoxin moxetumomab pasudotox (CAT-8015 or HA22) in patients with hairy cell leukemia. J. Clin. Oncol..

[57] Deng C., Xiong J., Gu X., Chen X., Wu S., Wang Z., Wang D., Tu J., Xie J. (2017). Novel recombinant immunotoxin of EGFR specific nanobody fused with cucurmosin, construction and antitumor efficiency in vitro. Oncotarget.

[58] Zhang C., Cai Y., Dai X., Wu J., Lan Y., Zhang H., Lu M., Liu J., Xie J. (2020). Novel EGFR-bispecific recombinant immunotoxin based on cucurmosin shows potent anti-tumor efficiency in vitro. Oncol. Rep..

[59] Cao L., Li Q., Tong Z., Xing Y., Xu K., Wang J.Y., Li W., Zhao J., Zhao L., Hong Z. (2020). HER2-specific immunotoxins constructed based on single-domain antibodies and the improved toxin PE24X7. Int. J. Pharm..

[60] Oliveira S., Schiffelers R.M., van der Veeken J., van der Meel R., Vongpromek R., van Bergen en Henegouwen P.M.P., Storm G., Roovers R.C. (2010). Downregulation of EGFR by a novel multivalent nanobody-liposome platform. J. Control. Release.

[61] van der Meel R., Oliveira S., Altintas I., Haselberg R., van der Veeken J., Roovers R.C., van Bergen en Henegouwen P.M.P., Storm G., Hennink W.E., Schiffelers R.M. (2012). Tumor-targeted Nanobullets: Anti-EGFR nanobody-liposomes loaded with anti-IGF-1R kinase inhibitor for cancer treatment. J. Control. Release.

[62] Talelli M., Rijcken C.J.F., Oliveira S., van der Meel R., van Bergen en Henegouwen P.M.P., Lammers T., van Nostrum C.F., Storm G., Hennink W.E. (2011). Nanobody-shell functionalized thermosensitive core-crosslinked polymeric micelles for active drug targeting. J. Control. Release.

[63] Talelli M., Oliveira S., Rijcken C.J.F., Pieters E.H.E., Etrych T., Ulbrich K., van Nostrum R.C.F., Storm G., Hennink W.E., Lammers T. (2013). Intrinsically active nanobody-modified polymeric micelles for tumor-targeted combination therapy. Biomaterials.

[64] Heukers R., Altintas I., Raghoenath S., De Zan E., Pepermans R., Roovers R.C., Haselberg R., Hennink W.E., Schiffelers R.M., Kok R.J. (2014). Targeting hepatocyte growth factor receptor (Met.) positive tumor cells using internalizing nanobody-decorated albumin nanoparticles. Biomaterials.

[65] Altintas I., Heukers R., van der Meel R., Lacombe M., Amidi M., van Bergen En Henegouwen P.M.P., Hennink W.E., Schiffelers R.M., Kok R.J. (2013). Nanobody-albumin nanoparticles (NANAPs) for the delivery of a multikinase inhibitor 17864 to EGFR overexpressing tumor cells. J. Control. Release.

[66] Dickens E., Ahmed S. (2018). Principles of cancer treatment by chemotherapy. Surgery.

[67] Oun R., Moussa Y.E., Wheate N.J. (2018). The side effects of platinum-based chemotherapy drugs: A review for chemists. Dalton Trans..

[68] Ding L., Gu W., Zhang Y., Yue S., Sun H., Cornelissen J.J.L.M., Zhong Z. (2019). HER2-Specific Reduction-Sensitive Immunopolymersomes with High Loading of Epirubicin for Targeted Treatment of Ovarian Tumor. Biomacromolecules.

[69] Liu T., Song P., Märcher A., Kjems J., Yang C., Gothelf K.V. (2019). Selective Delivery of Doxorubicin to EGFR+ Cancer Cells by Cetuximab-DNA Conjugates. Chembiochem.

[70] Rusch T., Bayry J., Werner J., Shevchenko I., Bazhin A.V. (2018). Immunotherapy as an Option for Cancer Treatment. Arch. Immunol. Ther. Exp..

[71] Vesely M.D., Schreiber R.D. (2013). Cancer immunoediting: Antigens, mechanisms, and implications to cancer immunotherapy. Ann. N. Y. Acad. Sci..

[72] Schreiber R.D., Old L.J., Smyth M.J. (2011). Cancer immunoediting: Integrating immunity’s roles in cancer suppression and promotion. Science.

[73] Dunn G.P., Old L.J., Schreiber R.D. (2004). The immunobiology of cancer immunosurveillance and immunoediting. Immunity.

[74] Lollini P.L., Cavallo F., Nanni P., Forni G. (2006). Vaccines for tumour prevention. Nat. Rev. Cancer.

[75] Kenderian S.S., Ruella M., Shestova O., Klichinsky M., Aikawa V., Morrissette J.J.D., Scholler J., Song D., Porter D.L., Carroll M. (2015). CD33-specific chimeric antigen receptor T cells exhibit potent preclinical activity against human acute myeloid leukemia. Leukemia.

[76] An N., Hou Y.N., Zhang Q.X., Li T., Zhang Q.L., Fang C., Chen H., Lee H.C., Zhao Y.J., Du X. (2018). Anti-Multiple Myeloma Activity of Nanobody-Based Anti-CD38 Chimeric Antigen Receptor T Cells. Mol. Pharm..

[77] Vacchelli E., Aranda F., Bloy N., Buqué A., Cremer I., Eggermont A., Fridman W.H., Fucikova J., Galon J., Spisek R. (2016). Trial Watch-Immunostimulation with cytokines in cancer therapy. Oncoimmunology.

[78] Nam J.S., Terabe M., Mamura M., Kang M.J., Chae H., Stuelten C., Kohn E., Tang B., Sabzevari H., Anver M.R. (2008). An anti-transforming growth factor beta antibody suppresses metastasis via cooperative effects on multiple cell compartments. Cancer Res..

[79] Henry K.A., Hussack G., Collins C., Zwaagstra J.C., Tanha J., MacKenzie C.R. (2016). Isolation of TGF-β-neutralizing single-domain antibodies of predetermined epitope specificity using next-generation DNA sequencing. Protein. Eng. Des. Sel..

[80] Maussang D., Mujic-Delic A., Descamps F.J., Stortelers C., Vanlandschoot P., Stigter-Van Walsum M., Vischer H.F., Van Roy M., Vosjan M., Gonzalez-Pajuelo M. (2013). Llama-derived single variable domains (nanobodies) directed against chemokine receptor CXCR7 reduce head and neck cancer cell growth in vivo. J. Biol. Chem..

[81] Weiner L.M., Dhodapkar M.V., Ferrone S. (2009). Monoclonal antibodies for cancer immunotherapy. Lancet.

[82] Pardoll D.M. (2012). The blockade of immune checkpoints in cancer immunotherapy. Nat. Rev. Cancer.

[83] Schadendorf D., Hodi F.S., Robert C., Weber J.S., Margolin K., Hamid O., Patt D., Chen T.T., Berman D.M., Wolchok J.D. (2015). Pooled Analysis of Long-Term Survival Data From Phase II and Phase III Trials of Ipilimumab in Unresectable or Metastatic Melanoma. J. Clin. Oncol..

[84] Eroglu Z., Kim D.W., Wang X., Camacho L.H., Chmielowski B., Seja E., Villanueva A., Ruchalski K., Glaspy J.A., Kim K.B. (2015). Long term survival with cytotoxic T lymphocyte-associated antigen 4 blockade using tremelimumab. Eur. J. Cancer.

[85] Sharma P., Sohn J., Shin S.J., Oh D.Y., Keam B., Lee H.J., Gizzi M., Kalinka E., de Vos F.Y.F.L., Ruscica D. (2020). Efficacy and Tolerability of Tremelimumab in Locally Advanced or Metastatic Urothelial Carcinoma Patients Who Have Failed First-Line Platinum-Based Chemotherapy. Clin. Cancer Res..

[86] Wan R., Liu A., Hou X., Lai Z., Li J., Yang N., Tan J., Mo F., Hu Z., Yang X. (2018). Screening and antitumor effect of an anti-CTLA-4 nanobody. Oncol. Rep..

[87] Tang Z., Mo F., Liu A., Duan S., Yang X., Liang L., Hou X., Yin S., Jiang X., Vasylieva N. (2019). A Nanobody Against Cytotoxic T-Lymphocyte Associated Antigen-4 Increases the Anti-Tumor Effects of Specific CD8 + T Cells. J. Biomed. Nanotechnol..

[88] Riella L.V., Paterson A.M., Sharpe A.H., Chandraker A. (2012). Role of the PD-1 Pathway in the Immune Response. Am. J. Transplant..

[89] Boussiotis V.A. (2016). Molecular and Biochemical Aspects of the PD-1 Checkpoint Pathway. N. Engl. J. Med..

[90] Buchbinder E.I., Desai A. (2016). CTLA-4 and PD-1 Pathways: Similarities, Differences, and Implications of Their Inhibition. Am. J. Clin. Oncol..

[91] Apolo A.B., Infante J.R., Balmanoukian A., Patel M.R., Wang D., Kelly K., Mega A.E., Britten C.D., Ravaud A., Mita A.C. (2017). Avelumab, an Anti-Programmed Death-Ligand 1 Antibody, in Patients with Refractory Metastatic Urothelial Carcinoma: Results from a Multicenter, Phase Ib Study. J. Clin. Oncol..

[92] Powles T., Park S.H., Voog E., Caserta C., Valderrama B.P., Gurney H., Kalofonos H., Radulović S., Demey W., Ullén A. (2020). Avelumab Maintenance Therapy for Advanced or Metastatic Urothelial Carcinoma. N. Engl. J. Med..

[93] Hellmann M.D., Ciuleanu T.E., Pluzanski A., Lee J.S., Otterson G.A., Audigier-Valette C., Minenza E., Linardou H., Burgers S., Salman P. (2018). Nivolumab plus Ipilimumab in Lung Cancer with a High Tumor Mutational Burden. N. Engl. J. Med..

[94] Paz-Ares L., Luft A., Vicente D., Tafreshi A., Gümüş M., Mazières J., Hermes B., Çay Şenler F., Csőszi T., Fülöp A. (2018). Pembrolizumab plus Chemotherapy for Squamous Non–Small-Cell Lung Cancer. N. Engl. J. Med..

[95] Horn L., Mansfield A.S., Szczęsna A., Havel L., Krzakowski M., Hochmair M.J., Huemer F., Losonczy G., Johnson M.L., Nishio M. (2018). First-Line Atezolizumab plus Chemotherapy in Extensive-Stage Small-Cell Lung Cancer. N. Engl. J. Med..

[96] Xian Z., Ma L., Zhu M., Li G., Gai J., Chang Q., Huang Y., Ju D., Wan Y. (2019). Blocking the PD-1-PD-L1 axis by a novel PD-1 specific nanobody expressed in yeast as a potential therapeutic for immunotherapy. Biochem. Biophys. Res. Commun..

[97] Wen B., Zhao L., Wang Y., Qiu C., Xu Z., Huang K., Zhu H., Li Z., Li H. (2020). Nanobodies targeting the interaction interface of programmed death receptor 1 (PD-1)/PD-1 ligand 1 (PD-1/PD-L1). Prep. Biochem. Biotechnol..

[98] Li S., Jiang K., Wang T., Zhang W., Shi M., Chen B. (2020). Hua, Z. Nanobody against PDL1. Biotechnol. Lett..

[99] Zhang F., Wei H., Wang X., Bai Y., Wang P., Wu J., Jiang X., Wang Y., Cai H., Xu T. (2017). Structural basis of a novel PD-L1 nanobody for immune checkpoint blockade. Cell Discov..

[100] Long L., Zhang X., Chen F., Pan Q., Phiphatwatchara P., Zeng Y., Chen H. (2018). The promising immune checkpoint LAG-3: From tumor microenvironment to cancer immunotherapy. Genes Cancer.

[101] Lecocq Q., Zeven K., De Vlaeminck Y., Martens S., Massa S., Goyvaerts C., Raes G., Keyaerts M., Breckpot K., Devoogdt N. (2019). Noninvasive Imaging of the Immune Checkpoint LAG-3 Using Nanobodies, from Development to Pre-Clinical Use. Biomolecules.

[102] Workman C.J., Cauley L.S., Kim I.J., Blackman M.A., Woodland D.L., Vignali D.A.A. (2004). Lymphocyte activation gene-3 (CD223) regulates the size of the expanding T cell population following antigen activation in vivo. J. Immunol..

[103] Grosso J.F., Kelleher C.C., Harris T.J., Maris C.H., Hipkiss E.L., De Marzo A., Anders R., Netto G., Getnet D., Bruno T.C. (2007). LAG-3 regulates CD8+ T cell accumulation and effector function in murine self- and tumor-tolerance systems. J. Clin. Investig..

[104] Yu X., Huang X., Chen X., Liu J., Wu C., Pu Q., Wang Y., Kang X., Zhou L. (2019). Characterization of a novel anti-human lymphocyte activation gene 3 (LAG-3) antibody for cancer immunotherapy. MAbs.

[105] He Y., Cao J., Zhao C., Li X., Zhou C., Hirsch F.R. (2018). TIM-3, a promising target for cancer immunotherapy. Onco. Targets Ther..

[106] Homayouni V., Ganjalikhani-Hakemi M., Rezaei A., Khanahmad H., Behdani M., Lomedasht F.K. (2016). Preparation and characterization of a novel nanobody against T-cell immunoglobulin and mucin-3 (TIM-3). Iran J. Basic Med. Sci..

[107] Chiba S., Baghdadi M., Akiba H., Yoshiyama H., Kinoshita I., Dosaka-Akita H., Fujioka Y., Ohba Y., Gorman J.V., Colgan J.D. (2012). Tumor-infiltrating DCs suppress nucleic acid-mediated innate immune responses through interactions between the receptor TIM-3 and the alarmin HMGB1. Nat. Immunol..

[108] Cai C., Xu Y.F., Wu Z.J., Dong Q., Li M.Y., Olson J.C., Rabinowitz Y.M., Wang L.H., Sun Y. (2016). Tim-3 expression represents dysfunctional tumor infiltrating T cells in renal cell carcinoma. World J. Urol..

[109] Clayton K.L., Haaland M.S., Douglas-Vail M.B., Mujib S., Chew G.M., Ndhlovu L.C., Ostrowski M.A. (2014). T cell Ig and mucin domain-containing protein 3 is recruited to the immune synapse, disrupts stable synapse formation, and associates with receptor phosphatases. J. Immunol..

[110] Sakuishi K., Apetoh L., Sullivan J.M., Blazar B.R., Kuchroo V.K., Anderson A.C. (2010). Targeting Tim-3 and PD-1 pathways to reverse T cell exhaustion and restore anti-tumor immunity. J. Exp. Med..

[111] Zhang D., Jiang F., Zaynagetdinov R., Huang H., Sood V.D., Wang H., Zhao X., Jenkins M.H., Ji Q., Wang Y. (2020). Identification and characterization of M6903, an antagonistic anti-TIM-3 monoclonal antibody. Oncoimmunology.

[112] Harjunpää H., Guillerey C. (2020). TIGIT as an emerging immune checkpoint. Clin. Exp. Immunol..

[113] Solomon B.L., Garrido-Laguna I. (2018). TIGIT: A novel immunotherapy target moving from bench to bedside. Cancer Immunol. Immunother.

[114] Callahan M.K., Postow M.A., Wolchok J.D. (2014). CTLA-4 and PD-1 pathway blockade: Combinations in the clinic. Front. Oncol..

[115] Wolchok J.D., Kluger H., Callahan M.K., Postow M.A., Rizvi N.A., Lesokhin A.M., Segal N.H., Ariyan C.E., Gordon R.A., Reed K. (2013). Nivolumab plus Ipilimumab in Advanced Melanoma. N. Engl. J. Med..

[116] Bjoern J., Lyngaa R., Andersen R., Hölmich L.R., Hadrup S.R., Donia M., Svane I.M. (2017). Influence of ipilimumab on expanded tumour derived T cells from patients with metastatic melanoma. Oncotarget.

[117] Takaya S., Saito H., Ikeguchi M. (2015). Upregulation of Immune Checkpoint Molecules, PD-1 and LAG-3, on CD4+ and CD8+ T Cells after Gastric Cancer Surgery. Yonago Acta Med..

[118] He Y., Yu H., Rozeboom L., Rivard C.J., Ellison K., Dziadziuszko R., Suda K., Ren S., Wu C., Hou L. (2017). LAG-3 Protein Expression in Non-Small Cell Lung Cancer and Its Relationship with PD-1/PD-L1 and Tumor-Infiltrating Lymphocytes. J. Thorac. Oncol..

[119] Mishra A.K., Kadoishi T., Wang X., Driver E., Chen Z., Wang X.J., Wang J.H. (2016). Squamous cell carcinomas escape immune surveillance via inducing chronic activation and exhaustion of CD8+ T Cells co-expressing PD-1 and LAG-3 inhibitory receptors. Oncotarget.

[120] Zuazo M., Arasanz H., Fernández-Hinojal G., García-Granda M.J., Gato M., Bocanegra A., Martínez M., Hernández B., Teijeira L., Morilla I. (2019). Functional systemic CD4 immunity is required for clinical responses to PD-L1/PD-1 blockade therapy. EMBO Mol. Med..

[121] Takano S., Saito H., Ikeguchi M. (2016). An increased number of PD-1+ and Tim-3+ CD8+ T cells is involved in immune evasion in gastric cancer. Surg. Today.

[122] Liu J., Zhang S., Hu Y., Yang Z., Li J., Liu X., Deng L., Wang Y., Zhang X., Jiang T. (2016). Targeting PD-1 and Tim-3 Pathways to Reverse CD8 T-Cell Exhaustion and Enhance Ex Vivo T-Cell Responses to Autologous Dendritic/Tumor Vaccines. J. Immunother..

[123] Zhou Q., Munger M.E., Veenstra R.G., Weigel B.J., Hirashima M., Munn D.H., Murphy W.J., Azuma M., Anderson A.C., Kuchroo V.K. (2011). Coexpression of Tim-3 and PD-1 identifies a CD8+ T-cell exhaustion phenotype in mice with disseminated acute myelogenous leukemia. Blood.

[124] Koyama S., Akbay E.A., Li Y.Y., Herter-Sprie G.S., Buczkowski K.A., Richards W.G., Gandhi L., Redig A.J., Rodig S.J., Asahina H. (2016). Adaptive resistance to therapeutic PD-1 blockade is associated with upregulation of alternative immune checkpoints. Nat. Commun..

[125] Zhou G., Sprengers D., Boor P.P.C., Doukas M., Schutz H., Mancham S., Pedroza-Gonzalez A., Polak W.G., de Jonge J., Gaspersz M. (2017). Antibodies Against Immune Checkpoint Molecules Restore Functions of Tumor-Infiltrating T Cells in Hepatocellular Carcinomas. Gastroenterology.

[126] Chauvin J.M., Pagliano O., Fourcade J., Sun Z., Wang H., Sander C., Kirkwood J.M., Chen T.H., Maurer M., Korman A.J. (2015). TIGIT and PD-1 impair tumor antigen–specific CD8+ T cells in melanoma patients. J. Clin. Investig..

[127] Ma L., Gai J., Qiao P., Li Y., Li X., Zhu M., Li G., Wan Y. (2020). A novel bispecific nanobody with PD-L1/TIGIT dual immune checkpoint blockade. Biochem. Biophys. Res. Commun..

[128] van de Water J.A.J.M., Bagci-Onder T., Agarwal A.S., Wakimoto H., Roovers R.C., Zhu Y., Kasmieh R., Bhere D., Van Bergen en Henegouwen P.M.P., Shah K. (2012). Therapeutic stem cells expressing variants of EGFR-specific nanobodies have antitumor effects. Proc. Natl. Acad. Sci. USA.

[129] Roovers R.C., Laeremans T., Huang L., De Taeye S., Verkleij A.J., Revets H., De Haard H.J., Van Bergen en Henegouwen P.M.P. (2007). Efficient inhibition of EGFR signaling and of tumour growth by antagonistic anti-EFGR nanobodies. Cancer Immunol. Immunother..

[130] Liu X., Newton R.C., Scherle P.A. (2011). Development of c-MET pathway inhibitors. Expert Opin. Investig. Drugs.

[131] Wen P.Y., Schiff D., Cloughesy T.F., Raizer J.J., Laterra J., Smitt M., Wolf M., Oliner K.S., Anderson A., Zhu M. (2011). A phase II study evaluating the efficacy and safety of AMG 102 (rilotumumab) in patients with recurrent glioblastoma. Neuro-Oncology.

[132] Okamoto W., Okamoto I., Tanaka K., Hatashita E., Yamada Y., Kuwata K., Yamaguchi H., Arao T., Nishio K., Fukuoka M. (2010). TAK-701, a humanized monoclonal antibody to hepatocyte growth factor, reverses gefitinib resistance induced by tumor-derived HGF in non-small cell lung cancer with an EGFR mutation. Mol. Cancer Ther..

[133] Huet H.A., Growney J.D., Johnson J.A., Li J., Bilic S., Ostrom L., Zafari M., Kowal C., Yang G., Royo A. (2014). Multivalent nanobodies targeting death receptor 5 elicit superior tumor cell killing through efficient caspase induction. MAbs.

